# Hypnogram-Driven Automatic Sleep Staging and a Quality-Index Assessment Through a Two-Stage LSTM-DNN Ensemble Learning Approach Using Multi-Biosignal Features for Sleep Disorder Detection

**DOI:** 10.3390/s26134091

**Published:** 2026-06-27

**Authors:** Roberto De Fazio, Matteo Paiano, Carolina Del-Valle-Soto, Ramiro Velazquez, Bassam Al-Naami, Paolo Visconti

**Affiliations:** 1 Department of Innovation Engineering, University of Salento, Road to Monteroni, 73100 Lecce, Italy; roberto.defazio@unisalento.it (R.D.F.); matteo.paiano@unisalento.it (M.P.); 2 Facultad de Ingeniería, Universidad Panamericana, Aguascalientes 20290, Mexico; rvelazquez@up.edu.mx; 3 Facultad de Ingeniería, Universidad Panamericana, Álvaro del Portillo 49, Zapopan 45010, Mexico; cvalle@up.edu.mx; 4 Department of Biomedical Engineering, Faculty of Engineering, The Hashemite University, Zarqa 13133, Jordan; b.naami@hu.edu.jo

**Keywords:** sleep scoring, EEG, EOG, PPG, two-stage DL algorithm, ensemble learning, hypnogram, sleep quality index

## Abstract

Sleep monitoring and analysis are essential for understanding overall health, improving sleep quality, and detecting potential disorders early. This study presents a multimodal approach for automatic sleep staging and quality assessment using a reduced set of bio-signals: a single electroencephalographic (EEG) lead (F4–F3), a single EOG lead, and the photo-plethysmographic (PPG) signal. The proposed methodology includes a hierarchical sleep staging classifier, an automatic sleep staging algorithm, and a subject-specific Sleep Quality Index (SQI) for objective sleep quality assessment. The 5-class sleep staging classifier employs a cascaded architecture of two sequential 3-class models (Wake-REM-NREM and N1-N2-N3), trained and tested on multimodal features derived from physiological signals (EEG, EOG, and PPG) of the BOAS (Bitbrain Open Access Sleep) dataset. The resulting 5-class classifier achieved 90.8% accuracy with a reduced memory footprint (3.14 MB). To assess subject-independent generalization and prevent data leakage between training and test sets, a Leave-One-Subject-Out (LOSO) validation was performed, confirming the robustness of the proposed classifier across unseen subjects. The classifier was subsequently integrated into an automatic sleep staging algorithm. Validation on 14 unseen subjects yielded accuracies ranging from 80.26% to 91.99% using heuristic post-processing rules, while a Hidden Markov Model (HMM)-based approach further improved performance, reaching a peak accuracy of 91.99%. The proposed SQI combines sleep-related metrics extracted from staging, considering multiple sleep aspects (i.e., duration, intensity, and continuity-fragmentation). A calibration strategy was proposed to customize the SQI based on sleep scoring parameters and the subjective quality score derived from sleep diaries and questionnaires (PSQI). This subject-specific strategy was validated on a public dataset, optimizing weights across multiple nights, followed by an independent test on a subsequent night and demonstrating strong alignment between the calculated SQI and the subjective sleep quality score (MAE = 10.81). Finally, the framework provides resource-efficient sleep staging and custom quality estimation, validating its readiness for practical, long-term sleep monitoring.

## 1. Introduction

Sleep is a fundamental physiological function essential for maintaining cognitive performance, physical health, and psychological well-being. Insufficient or poor-quality sleep is associated with cognitive impairments, reduced decision-making abilities, and an increased risk of cardiovascular diseases and mood disorders [1,2,3]. These issues become even more critical in high-demand operational contexts, such as space missions [4,5]. However, traditional polysomnography presents significant limitations, including its bulky, intrusive setup, the need for highly controlled environments, and its lack of suitability for long-term continuous monitoring [6,7]. These constraints make it impractical for space applications, where microgravity and operational constraints require compact and non-invasive solutions [8]. In this context, the SOMNIIA MONITOR project, funded by the Italian Space Agency (ASI), aims to develop a wearable system for continuous monitoring of sleep and other physiological parameters during space missions; specifically, a sensorized sleep mask capable of detecting a set of signals useful for staging and assessing sleep quality [9]. The sleep mask enables continuous, precise monitoring of biosignals and physiological parameters and can perform automatic sleep staging. It is equipped with multiple sensors for electroencephalographic (EEG) [10], electrocardiographic (ECG) signals [11], blood oxygen saturation (SpO_2_), and body temperature. In addition, two flexible piezoelectric sensors [12] are used for eye movement monitoring, while one is used to measure respiratory rate (RR). An inertial sensor is employed to detect body movements.

Sleep scoring in clinical settings is typically performed manually by trained medical personnel. Nevertheless, the quality of this scoring depends on the clinician’s experience, and therefore, it may vary depending on who performs it [13]. Furthermore, automated sleep staging tools can speed up the process and enable a more standardized, efficient sleep scoring procedure [14]. Therefore, another objective of the SOMNIIA MONITOR project is to develop a software platform capable of acquiring and storing physiological data, performing automatic sleep staging, and assessing overall sleep quality. The proposed research fits precisely into this context, focusing on the development of an automatic sleep classification algorithm based on a sleep model trained and tested with a multimodal feature set derived from EEG [15], electrooculographic (EOG), and photoplethysmographic (PPG) signals acquired via a sleep mask. The extracted features are used to design and validate a deep learning (DL) model for automatic sleep stage classification. To this end, the Bitbrain Open Access Sleep (BOAS) dataset [16] was used to train the proposed model. The dataset contains EEG, EOG, and PPG signals recorded with the Brain Quick Plus Evolution PSG system (Micromed) from 128 nights. For EEG, a bipolar frontal derivation (F4−F3) was considered, whereas for EOG, a single unipolar derivation was used. Lastly, PPG was processed to derive cardio-respiratory features. To ensure high-quality input data, preprocessing techniques, including artifact removal and filtering, were applied to the acquired signals. The model was evaluated using a multimodal feature set composed of EEG, EOG, and cardiorespiratory features extracted from the PPG signal, to which two feature reduction techniques were applied, namely minimum Redundancy Maximum Relevance (mRMR) and Principal Component Analysis (PCA).

A DL architecture based on Long Short-Term Memory (LSTM) networks was adopted for its effectiveness in modeling temporal dependencies in physiological time series. A two-stage ensemble classification strategy was implemented; firstly, the model performs classification by distinguishing among Wake (W), Rapid Eye Movement (REM), and Non-Rapid Eye Movement (NREM) phases (WRN classifier); the second stage refines NREM into N1, N2, and N3, producing a 5-class output. The resulting model achieved 90.8% test accuracy and a 3.14 MB memory footprint on the multimodal feature set.

Once developed, the classifier was integrated into an automatic sleep staging pipeline. The resulting algorithm, based on the previously trained and validated classifier, takes features extracted from a subject (external to the training dataset) as input over an entire night of sleep. The algorithm extracts and classifies sequences of features from the set of input features and generates, as output, the hypnogram corresponding to the entire night’s sleep, which is post-processed to increase its reliability. The algorithm was further evaluated on external subjects not included in the training and testing phases to assess its generalization to previously unseen data. Specifically, 14 subjects were selected from the BOAS dataset, excluding those used in the dataset construction phase.

Currently, sleep quality is often assessed using subjective methods, such as self-reported questionnaires, which rely on individuals’ perceptions of their sleep [17]. However, these approaches lack objective, quantitative measures that accurately reflect the restorative quality of sleep. This limitation motivates the need for objective indices derived from physiological signals and sleep staging results, enabling a more reliable and reproducible assessment of sleep quality [18]. To address these limitations, this work proposes and validates an objective Sleep Quality Index (SQI) based on parameters derived from polysomnographic sleep staging. The index combines metrics for total sleep time, sleep efficiency (SE), continuity, and fragmentation in a weighted mathematical formula. In particular, the considered parameters include Total Sleep Time (TST), time spent in the N3 stage, Wake After Sleep Onset (WASO), and the Sleep Fragmentation Index (SFI). Experimental validation was conducted on a public dataset (“Sleep Architecture in Response to a Late Evening Competition in Team-Sport Athletes”), based on a study by J.A. Vitale et al. at Istituto di Ricovero e Cura a Carattere Scientifico (IRCCS) Istituto Ortopedico Galeazzi (Milan, Italy) [19]. This dataset includes relevant physiological parameters and a subjective sleep quality index, enabling evaluation of the correlation between the proposed SQI and clinical assessments, as well as optimization of the index weights. Also, the proposed SQI requires a calibration phase to customize the weights to the evaluated subject. Thus, a calibration strategy was proposed to optimize a target function: the Pearson correlation between the SQI and the subjective sleep quality score reported in the dataset.

The main novelties of the proposed work are:

- Development of a two-stage model based on LSTM networks for automatic sleep staging. It was trained and tested on a multimodal feature set with features extracted from EEG, EOG, and PPG signals. Each stage has feature sequences as input and performs 3-class classification (Wake-REM-NREM and N1-N2-N3); thus, by combining the two classifiers in cascade, the overall model enables 5-class sleep staging.
- Rigorous cross-subject and cross-dataset validation: A Leave-One-Subject-Out (LOSO) cross-validation scheme is used alongside an independent cross-dataset validation utilizing the public ISRUC-Sleep dataset. By evaluating the multimodal framework on an entirely separate cohort that includes individuals with sleep disorders and pathologies, this step explicitly rules out potential data overfitting or statistical bleeding from data augmentation. This dual-validation approach establishes a highly realistic and robust foundation for the model’s clinical generalizability across unseen, pathological population profiles.
- Integration of the trained/tested model into an algorithm for automatic sleep staging and generating hypnograms to validate the model on subjects not included in the training/testing dataset. The algorithm also incorporates post-processing of the resulting hypnogram using heuristic rules to enhance its reliability in recognizing correct sleep stages, including a smoothing procedure for REM sequences and a mechanism to discard non-physiological phase shifts. This approach was benchmarked against a Hidden Markov Model (HMM) under both integer and artifact-cleaned signal conditions. This analysis provides a quantitative reference point, highlighting how standard probabilistic models compare with our deep learning architecture.
- A novel, customized, and weighted objective *SQI*, which combines metrics derived from the sleep staging process, belonging to multiple sleep domains (duration, intensity, continuity-fragmentation), and enables the model to overcome the existing indexes proposed in the literature that rely on a single sleep aspect. The proposed SQI requires a prior calibration phase to adapt the subject-specific weights to individual sleep characteristics and sensations/habits (quantified through a subjective SQI).

The remainder of this paper is organized as follows. Section 2 first explores sleep staging-derived parameters used for objective sleep quality assessment and then reviews existing approaches proposed in the literature. Section 3 describes the dataset used for training and testing the sleep staging model, the architecture of the proposed two-stage deep learning model, the automatic sleep staging pipeline, and the definition of the proposed SQI. Section 4 presents the classification model’s performance, the validation of the staging algorithm on external subjects, and the validation of the SQI. Finally, Section 5 discusses the results for the sleep classifier, the automatic staging algorithm, and the SQI.

## 2. Literature Review on Metrics and Methods for Quantifying the Sleep Quality

### 2.1. Analysis of Metrics Derived from Sleep Scoring for Quantifying the Sleep Quality

Sleep quality, although traditionally defined by polysomnography (PSG) as the gold standard [20], lacks a universally accepted definition and is primarily assessed through subjective metrics. Nevertheless, objective measures derived from sleep staging can effectively capture different aspects of sleep quality. In the literature, sleep quality is often assessed through self-reported indices reflecting individual satisfaction, correlated with PSG parameters, environmental and clinical factors, and sleep diaries that collect information on subjective perceptions. These instruments capture the perceptual experience of sleep but may conflate general dissatisfaction with sleep disturbances. In contrast, objective indicators allow analysis of aspects not captured by subjective measures alone. Different factors influence both perceived and measured sleep quality, including chronic diseases, anxiety, depression, lifestyle habits, caffeine, nicotine, and alcohol consumption, physical activity, age, and the presence of sleep disorders. Moreover, individuals may evaluate sleep quality using different criteria: some base their judgment on the feeling of well-being upon awakening, others rely on perceived experience in the night [21,22,23].

In this context, among the most widely used tools for subjective sleep assessment are standardized questionnaires, such as:

- The Pittsburgh Sleep Quality Index (PSQI), a self-report questionnaire used to assess sleep quality in clinical populations, consists of 19 items grouped into seven domains: sleep duration, sleep disturbances, sleep latency, daytime dysfunction, SE, subjective sleep quality, and use of sleep medication [24].
- The National Institutes of Health Patient-Reported Outcomes Measurement Information System (PROMIS) provides a uniform scale to assess sleep disturbance [25].
- The Epworth Sleepiness Scale (ESS), which evaluates daytime sleepiness [26].
- The Sleep Quality Scale, in which subjects rate their sleep quality over 7 days across five categories ranging from very poor to excellent [27].
- The Self-rating Sleep and Awakening quality scale (SSA), which comprises 20 items and yields three subscores (somatic complaints, subjective sleep quality, and subjective awakening quality) and an overall score reflecting the sleep experience [21].

Conversely, objective sleep quality assessment relies on several parameters derived from polysomnographic sleep staging that describe sleep architecture. These include:

- Duration-related indicators, such as *TST*, defined as the total time spent asleep during the night excluding wake periods, the duration of individual sleep stages (Wake, N1, N2, N3, REM), and Sleep Latency (*SL*), i.e., the time required to fall asleep;
- Intensity-related indicators, such as Deep Sleep Percentage (proportion of N3 sleep), EEG Delta Wave Amplitude, and Heart Rate Variability (HRV);
- Continuity-related indicators, the SE (ratio between total sleep time and time spent in bed), number of awakenings, and WASO (total time spent awake after sleep onset);
- Stability-related indicators, evaluated through sleep fragmentation index, transitions between NREM/REM stages compared to expected physiological patterns, and standard deviation of heart and respiratory rate;
- Frequency-related indicators, related to the recurrence of awakenings, average heart rate per sleep stage, and micro-arousal patterns, i.e., the distribution of brief arousals across sleep stages [21].

Table 1, reported in the American Academy of Sleep Medicine (AASM) manual, presents a standardized set of sleep-scoring-derived parameters that can serve as useful indicators for sleep quality assessment [28].

Among the objective parameters, sleep duration is correlated with perceived quality. In most cases, insufficient sleep is associated with poorer quality, while adequate rest tends to promote a positive perception. For this reason, the TST in ref. [29] is considered the primary indicator of sleep quality. In line with the National Sleep Foundation (NSF) guidelines [30], it is assumed that about 8 h of sleep represents an optimal condition, while shorter or longer durations are associated with a progressive reduction in quality.

In ref. [31], sleep onset latency (SOL), sleep fragmentation, sleep quality, sleep duration, and wakefulness were assessed in subjects exposed to continuous background noise overnight. The results of the study indicate that exposure to continuous noise may facilitate falling asleep and the continuity of sleep; therefore, a reduction in sleep onset latency and/or fragmentation could, in fact, translate into better overall sleep quality, longer sleep duration, and fewer awakenings. SOL was measured using various methods (self-reports, questionnaires, experimental observations) and, when explicitly defined, corresponds to the time interval between bedtime and the onset of sleep. Three main findings emerge from the literature: in several studies conducted on adult subjects, continuous noise appears to reduce SOL compared with baseline values; however, no statistical analyses have been reported to support this observation. Overall, continuous noise appears to reduce both SOL and sleep fragmentation. However, these findings are not yet supported by robust statistical evidence, and the available evidence is considered limited overall.

Studies conducted in ref. [22], involving numerous volunteers monitored via EEG for several nights, have shown that subjective sleep assessments are moderately correlated with objective metrics, with SE emerging as the most significant predictor of perceived sleep quality. Also, better sleep is reported after nights characterized by higher SE, lower SOL, reduced WASO, and longer N3 and REM sleep durations. The associations between subjective and objective sleep quality persist in intra-individual analyses; after nights characterized by poorer objective sleep parameters, the same subject tends to report poorer subjective sleep quality. However, there is no evidence that EEG power alone or the number of awakenings contribute to subjective sleep quality. Finally, interventions aimed at improving SE appear more likely to result in a perceived benefit than other strategies.

WASO is a key indicator of sleep continuity and its macrostructural fragmentation. Research in ref. [22] analyzed multi-night recordings and revealed significant night-to-night variability in WASO, consistent with findings from randomized clinical trials involving patients with insomnia. Such variability in objective sleep parameters, including WASO, may constitute a cardiovascular risk factor independent of sleep quantity or overall sleep quality alone. Furthermore, increases in WASO are associated with worse subjective ratings, both across individuals and within the same individual across nights, confirming the role of nocturnal continuity as a determinant of the sleep experience.

The role of sleep in cognitive health is widely recognized in the scientific literature. It is well established that sleep deprivation, nocturnal respiratory disorders, and fragmented sleep are associated with a decline in cognitive function. In particular, slow-wave sleep (SWS), corresponding to stage N3 and commonly referred to as deep sleep, plays a central role in memory consolidation processes, especially in healthy adults and individuals with mild cognitive impairment. A greater amount of SWS has been associated with better overall cognitive performance, with evident effects on executive functions, language, and processing speed [32]; conversely, a reduction in SWS is associated with executive difficulties and a decline in visuospatial learning. Physiologically, SWS is characterized by synchronized delta activity and reflects homeostatic sleep need, which is closely linked to cortical synaptic potentiation during wakefulness; moreover, slow-wave-rich sleep following a learning experience promotes better information retention. Overall, these data suggest that deep sleep is not merely a passive response to daytime activity but rather an active contributor to supporting and optimizing cognitive performance.

Sleep disorders are frequently associated with various types of nocturnal awakenings, which can negatively impact cardiovascular health and cognitive function. In particular, sleep fragmentation and nocturnal arousal are key factors in the development and chronicity of sleep disorders. Microarousals, sudden transitions to states of increased cortical activation, disrupt sleep continuity and may contribute to symptoms such as daytime sleepiness, reduced cognitive performance, and alterations in general well-being [33]. According to AASM guidelines, microarousals are characterized by an abrupt change in EEG frequency that lasts 3–15 s. Occurring in any sleep stage without full awakening, these events trigger the appearance of alpha, theta, or frequencies above 16 Hz (excluding the spindle band) and represent a transitional phase between sleep and wakefulness, or between REM and NREM sleep. Although some spontaneous arousals are part of normal sleep physiology, an excessive frequency of such events is associated with a decline in sleep quality and potential repercussions on neurocognitive and physical health.

In ref. [34], SFI 1 (Sleep Fragmentation Index 1) was introduced as a composite measure of sleep fragmentation, initially in patients evaluated for sleep-disordered breathing, showing a good correlation with the Micro-Arousal Index (MAI) and high reliability across different nights (Table 2). Subsequent studies evaluated its use in heterogeneous clinical samples, highlighting that in insomniac patients, SFI 1 is strongly correlated with wakefulness after falling asleep, whereas in patients with sleep-disordered breathing, it is associated with activation indices related to respiratory events. This result suggests that the index can capture the predominant pathophysiological feature across different disorders. Overall, SFI 1 emerges as a simple, rapid, and potentially useful tool in clinical practice, though it requires further validation in larger, more diverse samples.

In ref. [35], SFI 2 was calculated as the proportion of awakenings or transitions to the lightest sleep stage (N1) from deeper stages or REM sleep, expressed as a proportion of total sleep duration (Table 2). By ignoring extremely transient awakenings, the index may be less sensitive to subtle microvariations. Still, it has demonstrated good construct validity through correlations with age, periodic limb movement indices, apnea–hypopnea indices, and sleep staging characteristics. Furthermore, good test–retest reproducibility has been documented in the laboratory, which supports the reliability of the measure as an indicator of sleep stability disturbances. Overall, the SFI 2 represents a robust indicator of transitions to states of greater superficiality and, together with other parameters, contributes to the quantitative description of alterations in sleep continuity.

Table 2 presents the objective parameters described above, derived from polysomnographic staging, along with their definitions, physiological significance, and reference ranges, which are useful for quantitatively describing the various aspects of sleep.

**Table 2:** Representative metrics of the macro- and microstructure of sleep can be selected to develop an algorithm for objectively assessing sleep quality.

Parameter	Description	Physiological Meaning	Range
Total Sleep Time (TST)	Total time actually spent asleep during the night, excluding periods of wakefulness, considering a sleep staging in epochs of duration ∆*t*. $TST=\sum_{i=1}^{N}1\left\{stage\in \left[N1,N2,N3,REM\right]\right\}\cdot \Delta t$	It indicates the overall amount of actual sleep. This metric is useful, but not sufficient on its own to assess sleep quality because it does not reflect aspects such as sleep continuity, depth, or fragmentation [29].	6.4 h (typical) 3.7–8.5 h (min–max) [36]
Sleep Onset Latency (SOL)	Time between lights-off and the onset of sleep. $SOL=t_{first\;sleep}-t_{lights-off}$	Indicates ease of falling asleep. A short SOL indicates an easily relaxed nervous system and regular sleep [31].	0.4 h (typical) 0.1–2.1 h (min–max) [36]
Sleep efficiency (SE)	Percentage of time actually spent sleeping compared to total time spent in bed. $SE=\frac{TST}{TIB}\times 100,$ TIB: *Total In Bed* (tempo totale trascorso a letto).	High SE (>80%) implies fewer awakenings and more stable, continuous sleep, which promotes muscle and tissue recovery, cognitive function consolidation, and autonomic regulation. SE is strongly correlated with subjective assessments of sleep quality and remains one of the best predictors among objective metrics [22].	89.9% (typical) 67.2–97.6% (min–max) [36]
Wake After Sleep Onset (WASO)	Total time spent awake, from the onset of sleep until the end of the night, excluding the initial period before falling asleep. $\left.WASO=\sum min.\;awake\;after\;falling\;asleep\right.$	A high WASO indicates fragmented sleep and frequent interruptions. Frequent awakenings reduce the effectiveness of deep sleep (N3) and REM sleep, increasing physiological stress, heart rate, and blood pressure [37].	0.4 h (typical) 0.0–2.5 h (min–max) [36]
Percentage of time in stage N3 on the TST	Percentage of total sleep time spent in stage N3 (deep sleep) compared to TST. $\%N3=\frac{Tempo\;in\;N3}{TST}\times 100$	Reductions in the percentages of deep sleep (N3) and REM sleep, independent of TST, are associated with declines in cognitive function and increased subjective fatigue, highlighting the importance of depth patterns [32].	20.8% (typical) 10.2–28.7% (min–max) [36]
Arousal Index (ArI)	A microarousal is a transient episode of brain activation during sleep that lasts 3–15 s, without fully awakening the subject. The Arousal Index is the ratio of the number of microarousal events to the total sleep duration (TST).	Sleep continuity indicator. A high number of micro-arousals (>15–20/h) indicates fragmented sleep, therefore of reduced quality. They reduce the amount of REM and N3 sleep [38].	(typical values) 10.6 h^−1^ (age 10–20), 10.8 h^−1^ (age 21–30), 16.8 h^−1^ (age 31–40), 16.5 h^−1^ (age 41–50), 21.9 h^−1^ (age 51–60), 21.9 h^−1^ (age 61–70) [38]
SFI 1 (Sleep Fragmentation Index)	It is defined as: $SFI\;1=\frac{NSS+A}{TST},$ where NSS is the number of stage shifts (passage from one stage to another) during the entire night of sleep, and A is the number of awakenings after sleep onset.	A metric that describes, from a physiological perspective, sleep fragmentation (in addition to counting micro-arousals). The NSS parameter reflects the stability of the neurophysiological circuits that regulate sleep: a high NSS (>25) indicates unstable sleep, with difficulty maintaining deep stages (N3 and REM). The A parameter indicates the number of arousals during sleep, associated with cortical and autonomic activation. In such cases, an increase in sympathetic tone, heart rate, and blood pressure is observed, which disrupts the processes of memory consolidation and metabolic recovery. Therefore, this metric captures both microstructural instability (NSS) and macrostructural disruptions (A). Normalization with the TST allows the metric to be comparable across multiple subjects and makes the sum of NSS and A independent of sleep duration [34]. Low values (<24 h^−1^) indicate stable sleep and good continuity. High values (>40 h^−1^) are characteristic of fragmented sleep.	32.1 h^−1^ (typical) 24–40 h^−1^ (min–max) [34]
SFI 2 (Sleep Fragmentation Index)	It is defined as: $SFI\;2=\frac{n_{an1}}{TST},$ where $n_{an1}$ is the number of arousals or stage shifts to stage N1 from stages N2, SWS, or REM.	A metric that measures the number of transitions into unstable (N1) and shallow (W and N1) sleep states from deep sleep states (N2, SWS, or REM), relative to TST. Physiologically, N1 is the lightest sleep stage immediately following wakefulness. The idea is that each transition into the N1 state corresponds to a return to light sleep, i.e., a partial interruption of deep or REM sleep, even if it does not result in an awakening. A correlation between this metric and elevated systolic blood pressure while awake has been demonstrated [35].	4.8 h^−1^ (typical) 2.6–7 h^−1^ (min–max) [35]

### 2.2. Algorithms to Quantitatively Measure the Sleep Quality

The scientific literature proposes several objective indices that combine PSG-derived parameters to quantify sleep quality. In particular, ref. [39] introduces the “*Sleep Index*”, a sleep quality metric that integrates aspects of sleep architecture based on polysomnographic data, combining information on the temporal distribution of sleep stages and the stability of sleep structure. The index is designed to emphasize periods spent in more favorable sleep stages through appropriate weighting coefficients, while penalizing sleep fragmentation. The mathematical formulation of the index is given by:

(1) $$Sleep\;Index=\frac{\sum_{i=0}^{n}T_{i}\cdot W_{i}}{T_{o}\cdot NSS}$$

where $T_{i}$ represents the time spent in each sleep stage, $W_{i}$ the corresponding weights, $T_{o}$ the total sleep time, and *NSS* the number of stage shifts. Therefore, the index is proportional to the weighted sum of time spent in each sleep stage and inversely proportional to both the number of stage transitions and the total sleep duration.

Ref. [40] proposes the *Ru-SATED* index, a composite metric designed to comprehensively evaluate sleep quality by integrating six key dimensions: regularity (*R*), satisfaction (*S*), alertness (*A*), timing (*T*), efficiency (*E*), and duration (*D*). Regularity was estimated using the average Sleep Regularity Index, which measures the probability of being in the same state (sleep or wake) at two time points 24 h apart, averaged over a 6-week observation period (range 0–100). Sleep efficiency was computed as the ratio of total sleep time to the duration of the rest period, averaged across nights during the same period. Satisfaction was defined as the mean self-reported sleep quality from daily sleep diaries using a Likert scale ranging from 1 (“very poor”) to 5 (“very good”). Alertness was assessed using the 8-item PROMIS SRI (Sleep-Related Impairment) short form, which was the only dimension measured at the beginning of the study period. Timing was calculated as the average sleep midpoint over the observation period. It was considered optimal when it fell between 02:00 and 04:00. Sleep duration was defined as the average nightly sleep time over the 6 weeks, with values between 420 and 540 min (7–9 h) considered optimal. Each dimension was binarized, assigning 0 (unfavorable condition) or 1 (favorable condition). The *Ru-SATED score* is therefore computed as:

*Ru-SATED score* = *R* + *S* + *A* + *T* + *E* + *D* (2)

The score ranges from 0 to 6, with higher values indicating better overall sleep quality. The score was computed separately using sleep diary and actigraphy data. Based on the empirical distribution, a score ≥ 3 (approximately corresponding to the 25th percentile) was considered indicative of good sleep quality.

Ref. [41] proposes a sleep quality index based on objective measures collected through a multimodal acquisition system. The system integrates sensors, including a triaxial accelerometer and a pressure sensor, to monitor patient activity, heart rate, and body posture during sleep. The acquired signals were processed to extract relevant physiological parameters, including NREM sleep duration (*TNR*), total sleep time (*TST*), number of apnea episodes (*NAE*), and total time spent in the primary sleep posture (*PT*). Sleep quality is expressed by combining these parameters as follows:

(3) $$Sleep\;Quality=\frac{TNR}{TST}\alpha +\left(100-N_{AE}\right)\beta +P_{T}\cdot \gamma$$

where *α*, *β*, and *γ* are empirically determined weighting factors, equal to 0.5, 0.3, and 0.2, respectively, such that their sum equals 1.

In addition, mobile and home-based sleep monitoring devices, such as those developed by Zeo Inc., have been widely used to track physiological characteristics of sleep stages [42]. These systems enable the collection of objective parameters of an individual’s sleep cycle, which can be used to derive quantitative indices for sleep quality assessment. Zeo Inc. introduced the *ZQ* index, a sleep quality metric that incorporates key aspects of sleep physiology. The *ZQ* index is defined as:

(4) $$ZQ=\left(TST\times 1+DST\times 1.5+RST\times 0.5\right)-(TIW\times 0.5+\frac{ATS}{15})\times 8.5$$

where *DST* is the duration of deep sleep, *RST* is the duration of REM sleep, *TIW* is the total time awake during the night, and *ATS* is the number of awakenings. The *ZQ* index provides a numerical estimation of sleep quality by combining both positive and negative factors. In particular, *TST*, deep sleep, and REM sleep contribute positively with different weights, while wake time and the number of awakenings act as penalizing factors. The final score is scaled by an optimal average sleep duration, typically set to 8.5 h.

Although *ZQ* is a practical and intuitive metric, it has a significant limitation: it relies on a fixed optimal sleep duration that does not account for inter-individual variability. As a result, individuals with naturally shorter sleep requirements may receive artificially lower scores, thereby reducing the index’s reliability. To address this limitation, Cheng et al. propose an extended formulation that introduces a personalized sleep duration parameter, denoted as a_p_ [43]. The original *ZQ* formulation is adjusted by scaling each parameter’s contribution based on the ratio between the average optimal sleep duration a_m_ (set to 8.5 h) and the personalized one. The resulting sleep quality index is defined as:

(5) $${SQ}_{obj}=\left(TST\times {\left(\frac{a_{m}}{a_{p}}\right)}^{2}+DST\times {\left(\frac{a_{m}}{a_{p}}\right)}^{2}\times 1.5+RST\times {\left(\frac{a_{m}}{a_{p}}\right)}^{2}\times 0.5-TIW\times {\left(\frac{a_{m}}{a_{p}}\right)}^{2}\times 0.5+\frac{ATS}{a_{A}}\right)\times a_{p}$$

where $a_{m}$ is the average optimal sleep duration, $a_{p}$ is the personalized optimal sleep duration, and $a_{A}$ represents a personalized normalization factor for awakenings. This formulation enables a more individualized assessment of sleep quality by tailoring the index to each subject’s sleep needs, while also allowing the incorporation of subjective and population-based data from sleep databases to estimate parameters such as *DST* and *RST* based on demographic characteristics (e.g., age).

Sleep posture transitions have also been investigated as an indicator of sleep quality. In particular, Miwa et al. analyzed the time intervals between body position changes during sleep to discriminate between light sleep (N1 and N2) and *SWS* [44]. Specifically, sleep was classified as light or deep depending on whether the frequency of position changes exceeded or fell below a predefined threshold of 20 transitions. Based on this approach, the percentage of time spent in SWS was estimated and used to define a sleep quality metric referred to as the Sleep Quality Score (*SQS*), derived from the proportion of deep sleep (*S_D_*) over the total sleep period (*S*):

(6) $$SQS=\frac{S_{D}}{S}$$

To validate this metric, an experiment was conducted on a healthy male subject (29 years old) using a wearable monitoring device (SenseWear), which continuously recorded physiological signals for 372 days. A subset of 299 days was used to compute the evaluation index Q. The Pearson product-moment correlation coefficient between Q and sleep duration S was −0.28, indicating a weak negative correlation that was statistically significant at the 1% level. This result suggests that sleep quality, as measured by the proposed index, tends to decrease with increasing sleep duration. This observation is consistent with known sleep physiology, which indicates that the proportion of light sleep typically increases toward the end of the sleep period.

Sleep posture and body movements during sleep have also been investigated as indicators of sleep quality. Han et al. define the sleeping pose as a sequence of unconscious body movements involving trunk rotations between two static states, not considering isolated limb movements as posture changes [45]. Based on this definition, body positions are classified into four categories: supine, prone, left, and right. To detect and quantify these movements, the authors employ actigraphy, a non-invasive technique for monitoring rest–activity cycles based on acceleration signals. Specifically, body movements and posture transitions are inferred from acceleration data acquired through a triaxial accelerometer (e.g., embedded in wearable devices or smartphones). The acceleration vector magnitude *A* is computed from the three axes after removing the gravitational component, providing a measure of movement intensity associated with posture changes. Based on these measurements, the authors propose a sleep quality index (*S_Quality_*) defined as:

(7) $$S_{Quality}=A\times W$$

where *A* represents the normalized magnitude of the acceleration vector derived from actigraphy data, and *W* is a weight factor empirically assigned according to the detected sleeping position (e.g., *W_supine_ = 0, W_prone_ = 1, W_left and right_ = 0.5*). This approach enables the quantification of sleep quality by combining movement intensity with posture-related information, providing a simple yet effective metric based on unobtrusive sensing technologies. Table 3 summarizes the sleep quality indices identified in the literature, along with the corresponding parameters used to compute them.

Comparison of the sleep quality indices reported in the literature (Table 3) shows that existing approaches typically rely on a limited set of variables, ranging from 2 (SQS, SQuality) to 7 (SQobj), and generally do not cover all the key dimensions of sleep quality, namely duration, intensity, continuity, stability, and frequency. Furthermore, several indices rely on empirically defined weighting factors or focus on a single aspect of sleep quality, such as sleep architecture, respiratory events, or body movements. As a result, they may provide only a partial representation of the overall sleep process. In contrast, the proposed objective sleep quality index (described in Section 3.5) integrates four complementary parameters that collectively capture all five dimensions considered in this study. Therefore, the proposed metric provides a more comprehensive representation of sleep quality. Moreover, the index is integrated into the proposed sleep staging pipeline (described in Section 3.4), as all the parameters required for its computation are automatically extracted from the sleep stage classification output. Therefore, objective sleep quality assessment can be performed directly after automatic sleep staging without requiring additional sensing modalities or post-processing procedures.

## 3. Materials and Methods

This section provides an overview of the steps taken to develop the sleep staging ensemble-learning model for sleep phase classification, which was trained and tested using a multimodal, optimized feature set extracted from a reduced set of biosignals [46]. Following this, the developed model was integrated into a custom pipeline, which receives as input the features of a subject over a full night’s sleep, extracts the feature sequences, classifies them into the typical 5-class scoring (Wake, REM, N1, N2, and N3) to obtain the raw hypnogram, and post-processes this last to detect non-physiological transitions and smooth out spurious predictions. Finally, this section defines a new SQI, taking into account multiple domains of sleep (duration, intensity, continuity, degree of fragmentation), thus addressing the main limitations of the existing sleep quality assessment methods, which are typically based on subjective evaluation from the subject or, when based on objective metrics, consider just single aspects of sleep. The section also describes the validation dataset, the procedure for subject-specific weight optimization, and the computation of the corresponding SQI values. In this way, a customized SQI is obtained for each subject.

### 3.1. Feature Extraction and Selection from the Signal Belonging to the BOAS Dataset

The proposed work uses the BOAS dataset, which includes 128 nights of simultaneous recordings from a clinical PSG system and a wearable Bitbrain EEG headband (manufactured by Bitbrain Co., Zaragoza, Spain), in healthy subjects. This dataset enables direct comparison between gold-standard PSG measurements and a more practical wearable EEG solution based on forehead electrodes.

Sleep stages were annotated on PSG data by three expert scorers following AASM guidelines using 30 s epochs, with a fourth expert generating consensus labels to reduce inter-scorer variability. The stages include Wake (0), N1 (1), N2 (2), N3 (3), REM (4), while disconnections (8) and artifacts (−2) were also labeled. These consensus labels were then transferred to the corresponding wearable EEG recordings.

**Figure 1:**
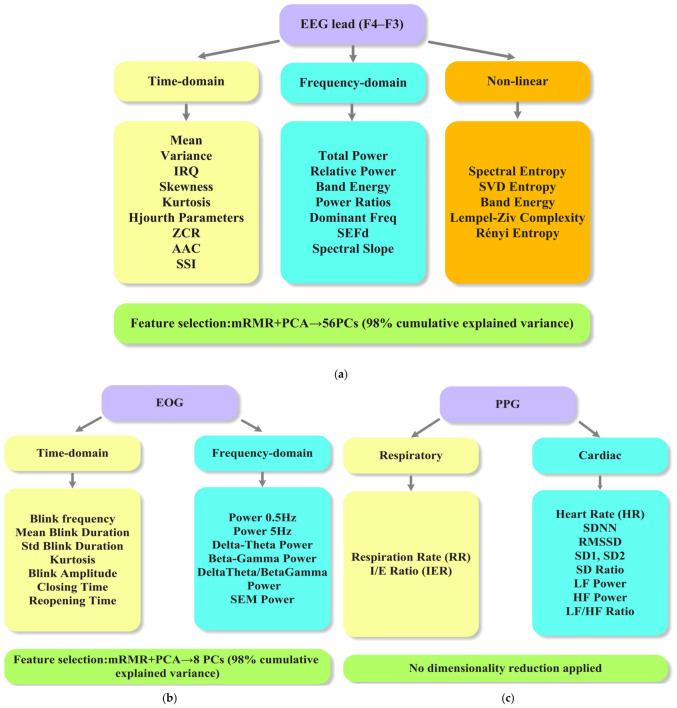
Block diagrams related to the extraction and optimization of the features from the different biosignals: (**a**) EEG, (**b**) EOG, and (**c**) PPG signals.

For the proposed work, a reduced set of biosignals was selected for use in combination with a wearable device to minimize the number of signals acquired, thereby avoiding hindrance to user movements and skin irritation [47,48]. In detail, an EEG frontal lead (F4−F3), an EOG derivation, and a PPG signal were considered for developing the sleep staging classifier, ensuring simplicity and reliability of acquisition in the face area. Features were extracted from the EEG, EOG, and PPG signals of 25 subjects from the BOAS dataset, totaling 183 h of sleep (131.401 non-overlapping 5 s epochs). Subjects were chosen based on the availability of all three signals (frontal EEG (F4–F3), single-channel EOG, and PPG) to ensure consistency. Before feature extraction, a signal quality assessment was performed to remove segments affected by artifacts, noise, or saturation. Additionally, epochs labeled as “−2” (artifacts) or “8” (disconnections) were excluded. EEG signals were preprocessed using band-pass filtering (0.2–44 Hz) and a 50 Hz notch filter, then segmented into 5 s epochs. Each epoch inherited the sleep stage label of its corresponding 30 s segment, and features were extracted accordingly. In particular, an initial feature set of 100 features was extracted, belonging to the time, frequency, and non-linear domains (Figure 1a). The use of 5 s sub-epochs inheriting the label of the corresponding 30 s epoch was a deliberate design choice to enhance the temporal granularity of the input feature sequences [49,50,51]. The proposed hierarchical sleep staging architecture uses LSTM networks to capture the complex dynamics of cardiorespiratory and EEG microarchitecture. To do this effectively, the model evaluates input feature sequences composed of 10 consecutive 5 s epochs, which represent a continuous 50 s window. Using 30 s epochs directly would limit each input sequence to only 1–2 time steps, which is insufficient for the subsequent LSTM layers (128 and 64 units) to model temporal dependencies adequately. We acknowledge that this approach may introduce some label noise into the feature sequences, particularly near stage transitions. However, we considered this trade-off acceptable given the substantial benefit of enhanced temporal granularity for sequence modeling.

EOG signals were band-pass filtered (0.5–10 Hz) and normalized. Blink detection was performed using a dynamic threshold based on signal statistics, identifying supra-threshold regions as candidate events. Valid blinks were selected based on amplitude, slope, and minimum duration (≥100 ms). Additional temporal features, such as eye closure and reopening times, were derived from zero-crossings and local extrema. Finally, the signal was segmented into 5 s epochs, labeled consistently with the EEG, and relevant blink-related features were extracted (Figure 1b).

Furthermore, respiratory features were extracted from the PPG signal by first computing the signal’s respiratory envelope using the Hilbert transform, followed by 8th-order band-pass filtering (0.1–0.5 Hz) to isolate respiration-related oscillations. The resulting signal was divided into non-overlapping 30 s windows. Within each window, respiratory cycles were identified using an adaptive threshold set to 70% of the signal RMS, allowing detection of inspiratory peaks and expiratory troughs. Respiratory cycles were defined as intervals between consecutive troughs, from which RR and inspiratory-to-expiratory ratio (IER) were computed (Figure 1c). These features were averaged per window and aligned with sleep stages. To match the temporal resolution of EEG and EOG features, each sample was repeated 6 times, yielding a 5 s resolution.

Cardiac features were extracted by isolating the cardiac component of the PPG using an 8th-order band-pass filter (0.5–4 Hz). The filtered signal was segmented into 30 s windows, and heartbeats were detected as positive peaks using an adaptive threshold set to 25% of the RMS. Additional constraints included a minimum peak distance of 0.3 s and a prominence threshold equal to the adaptive level. RR intervals were computed as the time difference between successive peaks. From these intervals, cardiac features were derived, averaged per window, and aligned with sleep stages (Figure 1c). As with respiratory features, the data were replicated six times to achieve a 5 s temporal resolution.

Afterward, the dimensionality of the EEG and EOG feature sets was reduced through a two-step procedure combining mRMR and PCA (Figure 1a,b). First, the mRMR algorithm ranked features based on their relevance to sleep-stage classification and their redundancy with the remaining features. A score threshold of 0.01 was applied, reducing the EEG feature set from 100 to 90 and the EOG feature set from 14 to 9. Subsequently, PCA was applied to the selected features. The number of principal components was determined by retaining 98% of the cumulative explained variance, resulting in 56 PCs for EEG and 8 PCs for EOG. This threshold provided the best trade-off between dimensionality reduction and information preservation, while also yielding the highest sleep-staging performance during model development. The retained PCs preserved most of the informative content of the original feature sets, limiting information loss to approximately 2% of the total variance. Since both mRMR ranking and PCA are deterministic procedures applied to the same dataset and preprocessing pipeline, the feature selection process is fully reproducible. Furthermore, the selected PCs consistently represented the most informative feature combinations identified after mRMR filtering, supporting the stability of the dimensionality reduction process. On the contrary, on the PPG feature set, no dimensionality reduction was applied (Figure 1c). While linear techniques like PCA do not explicitly capture non-linear interactions among the raw features, it is intentionally integrated into the pipeline to eliminate highly collinear features, isolate orthogonal components containing maximum variance, and mitigate the risk of overfitting caused by the high dimensionality of the hand-crafted feature space. Crucially, the preservation and modeling of complex, non-linear cross-channel interactions and non-linear cardiorespiratory temporal dynamics are not discarded; rather, they are systematically deferred to the subsequent LSTM and DNN layers. The recurrent units, equipped with non-linear activation functions (e.g., tanh and sigmoid gates), are mathematically optimized to reconstruct and learn high-level non-linear feature representations from orthogonalized low-dimensional projections provided by PCA.

### 3.2. Structure of the Two-Stage DL Model for Sleep Staging

**Figure 2:**
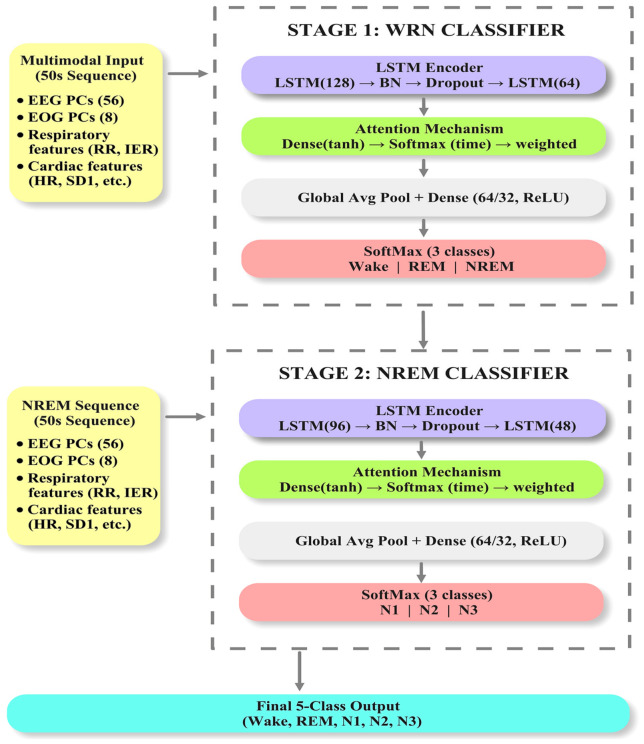
Two-stage LSTM-based sleep staging framework; stage 1 classifies 50 s multimodal sequences into Wake, REM, or NREM. Stage 2 refines NREM sequences into N1, N2, and N3 sub-stages.

The proposed sleep staging model is a two-stage deep learning framework using LSTM networks with an attention mechanism. The first stage, called WRN-classifier, processes multimodal input sequences (EEG and EOG PCs, respiratory and cardiac features from PPG), and assigns them to one of three sleep stages: Wake, REM, NREM. In the second stage, only the sequences labeled as NREM are analyzed by a dedicated classifier that differentiates among N1, N2, and N3 sub-stages. This hierarchical approach enables classification among the five sleep stages (Wake, REM, N1, N2, N3), simplifying the problem into two manageable tasks, improving class balance, and allowing each model to specialize, thus enhancing overall performance. Both classifiers share a similar structure: the WRN model uses LSTM layers with 128 and 64 units, and the NREM ones with 96 and 48 units (Figure 2). Each LSTM layer is followed by batch normalization and dropout to improve generalization and training stability. An attention mechanism is applied to LSTM outputs, using a tanh-activated dense layer followed by a temporal softmax to compute attention weights that emphasize the most informative time steps through element-wise multiplication.

The resulting features are then aggregated via global average pooling and passed through two fully connected layers (64 and 32 neurons, ReLU activation) with batch normalization and dropout, followed by a softmax layer producing the three-class output: Wake/REM/NREM for the WRN classifier and N1/N2/N3 for the NREM classifier.

### 3.3. Dataset Composition and Partitioning for Model Training and Testing

The dataset consists of 131,401 labeled 5 s epochs, distributed among Wake (13,143), REM (26,210), and NREM (92,048). Within NREM, the classes are highly imbalanced: 2887 N1, 83,635 N2, and 5526 N3 epochs, with the dominance of N2 and imbalance across stages.

To model temporal dependencies, epochs were grouped into 50 s sequences using a sliding-window approach (10 consecutive epochs). For the WRN classifier, overlapping windows (stride = 5) generated 26,279 sequences, split into 80% for training and 20% for testing. For the NREM classifier, only NREM epochs were retained and grouped using non-overlapping windows (stride = 10), yielding 9204 sequences divided into training (70%), validation (15%), and test (15%) sets. Each sequence was labeled via majority voting. Dataset partitioning was performed at the sequence level using stratified sampling, with a fixed random seed set to 42 to ensure reproducibility of the experimental setup. To mitigate class imbalance, data augmentation was applied exclusively to the training sets after dataset random partitioning, ensuring that no augmented samples were present in the validation or test sets, thereby minimizing the risk of statistical leakage.

**Figure 3:**
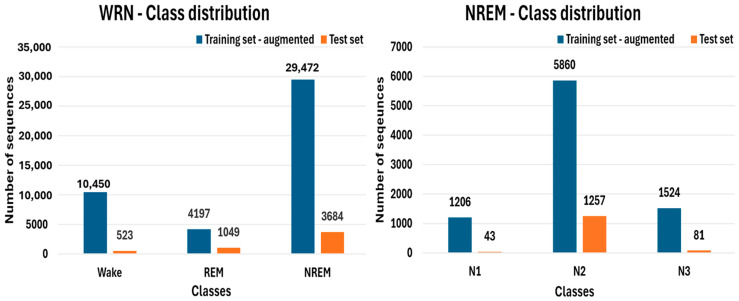
Distribution of training (blue) and test (orange) sets used for training and testing the WRN (**a**) and NREM (**b**) models.

Augmentation included noise addition, scaling, temporal shifts, masking, and amplitude attenuation. Minority classes were heavily oversampled: Wake (×5) and NREM (×3) for WRN, and N1 (×6) and N3 (×4) for NREM, increasing training sizes to 58,855 and 6442 sequences, respectively. Features were normalized using a StandardScaler (Python ver. 3.14, IDE PyCharm ver. 2025.2.3) fitted on the original training data. To further reduce bias toward majority classes, both models used a class-weighted loss (Balanced Crossentropy), assigning higher weights to underrepresented classes (especially N1 and N3 in NREM) to emphasize their contribution during training. Partially overlapping windows (stride = 5) were used with the WRN classifier to reduce class imbalance and increase the number of training samples from the small cohort. To reduce the risk of statistical leakage and artificial performance inflation, data augmentation was applied only after dataset partitioning, and model performance was assessed on non-augmented test sequences, even though this approach introduces some similarity between adjacent sequences. Additionally, it should be noted that the suggested model uses a feature-level abstraction to stratify the random assignment of windows during the training/testing split and to minimize temporal dependencies, thereby reducing the likelihood that nearby windows would fall into the same partition. For the NREM classifier, non-overlapping windows ensured that sequences had no temporal overlap. The final hierarchical system was evaluated on a test set of 2953 sequences, obtained by combining the WRN and NREM test sets. Figure 3 shows the class distribution: augmentation is applied to the training set, though it is not applied to the test set, for the WRN (a) and NREM (b) models. To further assess model generalizability, additional validation experiments were conducted on an independent cohort of 14 subjects from the BOAS dataset, as described in Section 4.2. Moreover, external validation was performed on subjects from a different publicly available sleep dataset, as reported in the Section Validation of the Staging Algorithm on Subjects of the ISRUC Datasets, providing an additional benchmark for evaluating the robustness of the proposed model.

### 3.4. Pipeline for Automatic Sleep Staging Based on the Trained and Tested Sleep Staging Classifier

After training and testing the classification model, the automatic staging algorithm was implemented. Specifically, the previously developed and trained hierarchical models (WRN and NREM), exported in Keras format, were loaded into a Python (ver. 3.14) inference environment to perform automatic classification on subjects not used during the ensemble learning model training phase. After describing the pipeline used to load and process data from the two models, the algorithm’s results are presented as confusion matrices, hypnogram comparisons with physician-annotated hypnograms, and accuracy metrics, obtained by testing the system on subjects not used during the training and testing phases. The classification algorithm implemented in the sleep stage estimation pipeline operates on data derived from preprocessed physiological signals (EEG, EOG, and PPG), represented as tabular features organized into 5 s epochs. The system architecture relies on two LSTM models, called WRN (Wake/REM/NREM) and NREM, which were previously trained and subsequently exported to the Keras format. The models are loaded into the algorithm at runtime using the library’s deserialization capabilities, restoring the architecture, learned weights, and training configurations. To implement this step, the previously saved models and scalers are loaded as shown in the following code snippet.

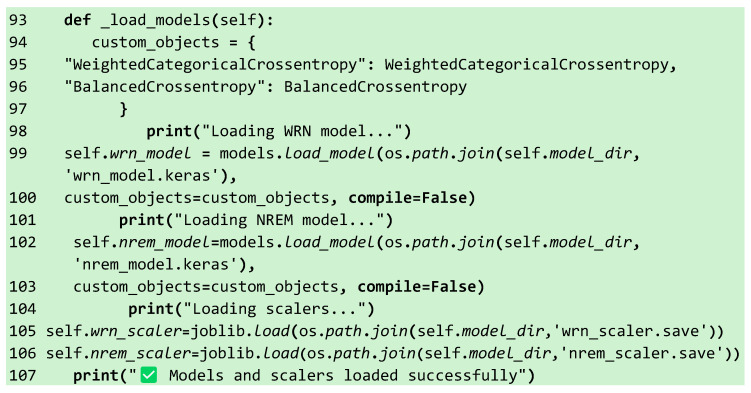



The WRN model is the first decision-making layer; it takes pre-processed time sequences and classifies each as Wake, REM, or NREM. If the prediction matches the NREM one, the same sequence is fed into the second NREM model, which discriminates between the N1, N2, and N3 stages. This hierarchical approach allows the problem to be divided into two levels, improving the overall robustness and classification accuracy.

**Figure 4:**
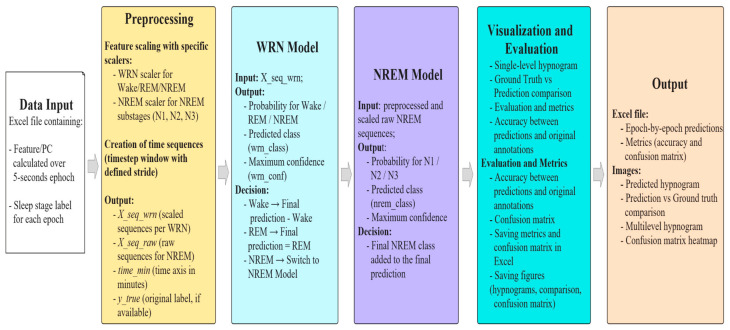
Block diagram of the sleep classification algorithm pipeline based on previously trained and tested staging models.

The preprocessing pipeline is designed to transform the raw biosignal data into structured time sequences of scaled and normalized features, optimized for direct use in the WRN and NREM classification models (Figure 4). The pipeline’s input consists of Excel files containing the complete set of features/principal components (PCs) computed over 5 s epochs, along with the corresponding sleep stage labels. Specifically, the features extracted from EEG, EOG, and PPG biosignals using the mRMR algorithm were applied to the EEG and EOG signals to retain the most informative variables and reduce redundancy. The selected features were then normalized using z-scores, with mean and standard deviation calculated from the training and test datasets, thereby ensuring statistical consistency between the training and inference phases. Then, the selected principal components were calculated from the normalized features to maintain a cumulative variance of 98%. For the PPG signals, however, the features (relating to the respiratory and cardiac components) previously selected during the dataset construction phase were extracted.

The features are scaled using scalers trained separately for the WRN (Wake/REM/NREM) and NREM models to normalize value distributions and reduce the impact of outliers or differences between subjects. Subsequently, the epochs are grouped into sequences of a length defined by the “timesteps” parameter (10 epochs in this case), with a “stride” (i.e., an overlap of 5 epochs) that creates overlapping windows, ensuring temporal continuity in the predictions and allowing the models to exploit the sequential dynamics of the data. For each sequence, the last epoch is considered representative for alignment with the original annotations during evaluation. The WRN model is applied to the preprocessed sequences to classify each window as Wake, REM, or NREM. The sequence generation procedure is implemented as shown in the following code snippet.

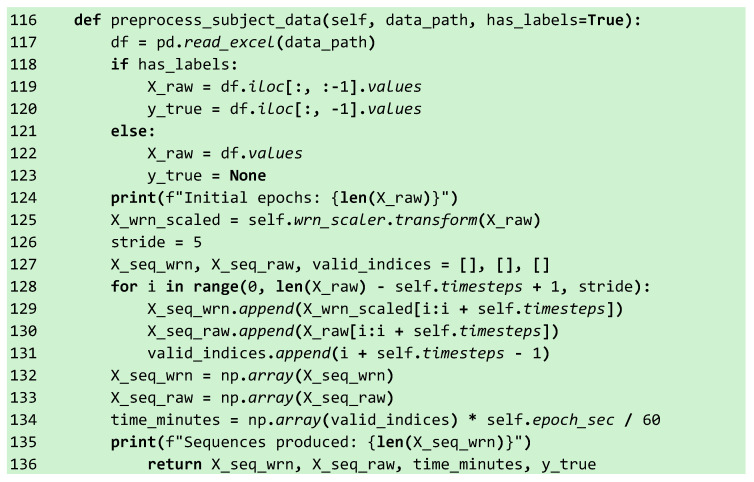



When the prediction indicates NREM, the NREM model is used to distinguish between the N1, N2, and N3 sub-stages. For each sequence, the predicted class and its associated confidence score, as well as the model’s maximum probability, are calculated. The hierarchical classification procedure is shown in the following code snippet.

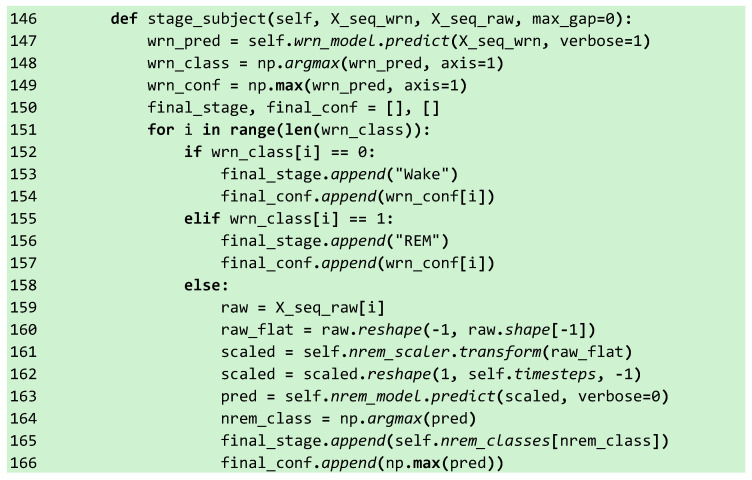



**Figure 5:**
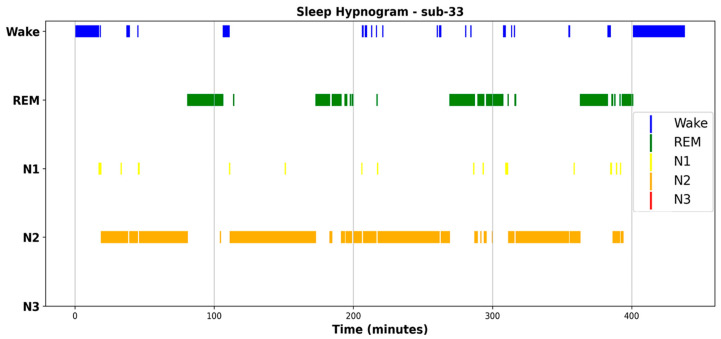
Example of a hypnogram generated by the developed sleep staging algorithm.

This hierarchical approach reduces overall classification error by focusing on the simplest task at the first level and delegating the finer distinction between deeper sleep stages to the second model. To improve the temporal consistency of predictions and avoid non-physiological fragmentation, a REM-stage propagation filter is applied at the sequence level. Specifically, after hierarchical classification, the indices corresponding to sequences labeled as REM are identified; if there are a limited number of intermediate sequences (less than or equal to the “max_gap” parameter) between two consecutive REM sequences, those sequences are reclassified as REM. This mechanism is applied exclusively to REM episodes because, during empirical analysis, REM predictions exhibited greater temporal instability and a greater tendency to appear as fragmented or isolated segments than those in other sleep stages. This mechanism allows for bridging short interruptions, potentially due to model uncertainties, reduces the artificial fragmentation of REM episodes, and preserves the temporal coherence typical of sleep architecture. Subsequently, an additional logical filter is applied to prevent transitions deemed physiologically unlikely. In particular, direct transitions between stage N3 (deep sleep) and the Wake stage in both directions are discarded. If the model predicts a transition from stage N3 to the Wake stage, the current sequence remains in N3; similarly, if it predicts a transition from the Wake stage to N3, the sequence is reclassified as Wake. These transitions are considered physiologically implausible because, according to established sleep architecture, transitions between deep sleep and wakefulness occur progressively through intermediate stages (N2 and N1) rather than occurring directly. This constraint is based on the physiological structure of sleep, according to which transitions between deep sleep and wakefulness generally occur gradually through intermediate stages (N1 and N2) [52], thereby making the final hypnogram more clinically realistic. The application of these post-processing rules thus introduces temporal regularization downstream of the model’s classification, integrating the predictive power of LSTM models with physiological constraints. For visualization and evaluation, the pipeline generates single-level hypnograms, in which each epoch is represented by a color corresponding to the predicted stage and temporally aligned to the recording minutes. The hypnograms are constructed such that the Y-axis follows the natural order of the stages, with Wake at the top and N3 at the bottom, ensuring an intuitive representation of sleep depth. An example of the generated hypnogram is shown in Figure 5.

**Figure 6:**
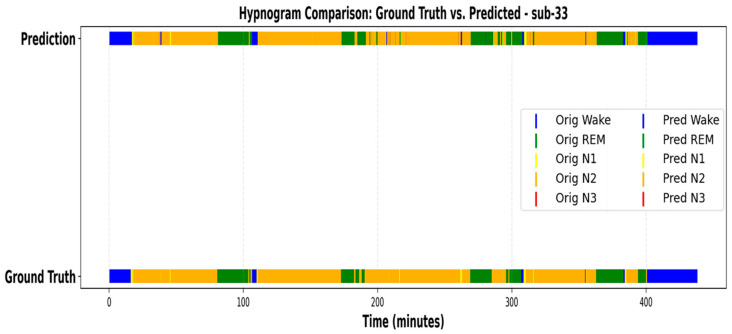
Comparison of the hypnograms generated by the developed sleep staging algorithm and the ground truth represented by the sleep staging data in the dataset for the subject considered.

The comparison between predictions and original annotations is presented in a plot with two overlaid subplots sharing an X-axis converted to minutes, with the first subplot showing the ground truth (i.e., the staging reported by the medical experts in the dataset). The second subplot represents the model’s predictions, providing a clear visual representation of the temporal alignment between predictions and the reference (Figure 6). The ground truth is represented by the annotations associated with the subject, obtained from the scoring by the three specialized physicians, with the agreement of a fourth physician, and reported in the last column of the Excel file uploaded by the model. The annotations are extracted from the file and aligned with the sequence series obtained from the features to evaluate the performance of the sleep staging algorithm.

**Figure 7:**
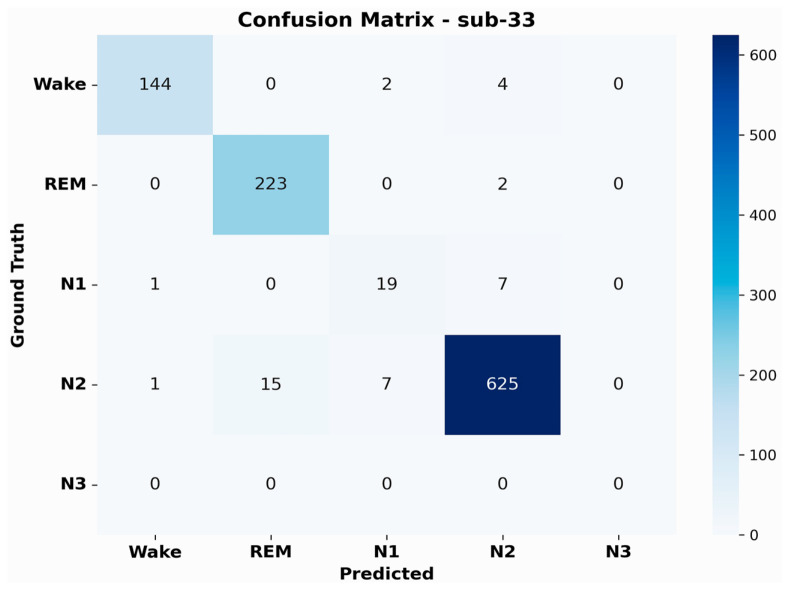
Confusion matrix comparing the predictions (in terms of sequences) provided by the sleep staging algorithm with the ground truth represented by the dataset’s annotations for each subject.

The quantitative evaluation includes overall accuracy and the confusion matrix, which is saved in an Excel file along with the accuracy, ensuring the traceability of the results for subsequent analyses or comparisons between subjects (Figure 7).

This algorithm integrates advanced preprocessing techniques, hierarchical models with deep neural networks, custom loss functions designed to handle class imbalance and temporal continuity in the data, and accurate visualization and evaluation tools. The combination of these elements enables reliable predictions of sleep stages, with clear visual representation and detailed quantitative metrics, making the system suitable for both clinical trials and neuroscientific research. The focus on class balance, secure model serialization, and temporal alignment of epochs ensures the robustness and reproducibility of results on future data, enabling in-depth analyses of sleep patterns at both the individual and population levels. The Python script of the sleep staging pipeline is available in the Supplementary Materials (File S2).

### 3.5. Definition of a New Objective Sleep Quality Index (SQI) Based on Sleep Scoring Data

Similar to the scientific studies described in Section 2.2, which propose sleep quality indices derived from polysomnographic sleep staging, this study introduces a comprehensive and objective indicator of overall nighttime sleep quality. The proposed Sleep Quality Index (SQI) is obtained as a weighted combination of several parameters derived from sleep staging, following the general approach adopted in the literature [20,22,23,24]. However, alternative approaches estimate sleep quality from wearable-based measurements rather than polysomnographic data. In particular, one method relies on a multisensor device that combines actigraphy with additional physiological signals [44], while another relies solely on actigraphy-derived motion and posture features extracted from accelerometer signals [45]. The *SQI* is given by the weighted sum of four terms that take into account the duration, continuity (degree of fragmentation), and intensity of sleep:

(8) $$SQI\;[\%]=\left[w_{1}\times \frac{TST}{{TST}_{max}}+w_{2}\times \frac{T_{N3}}{T_{N3max}}+w_{3}\times \left(\frac{1}{1+\frac{WASO\;\left[h\right]}{1h}}\right)+w_{4}\times \left(\frac{1}{1+\frac{SFI2}{TST}}\right)\right]\times 100$$

Specifically, the meanings of the various terms are as follows:

- $\frac{TST}{{TST}_{max}}$, takes into account total sleep time (TST) normalized to the maximum TST value (${TST}_{max}$), which depends on multiple factors (gender, age, etc.); for an adult male (18–29 years old), ${TST}_{max}$ can be set at 8.5 h [36];
- $\frac{T_{N3}}{T_{N3max}}=\frac{T_{N3}[min]}{{0.29\times TST}_{max}\;[min]}$, takes into account sleep intensity, i.e., its quality and restorative capacity. A reduced value of time spent in stage N3 implies greater vulnerability to sleep fragmentation. The value of $T_{N3}$ must be normalized to its maximum value ($T_{N3max}$), which depends on the patient’s age group; for an adult user (18–29 years), the value of $T_{N3max}$ is 28–29% of TST [36];
- $\left(\frac{1}{1+\frac{WASO\;\left[h\right]}{1h}}\right)$, accounts for sleep continuity, in particular, the total time the subject remains awake after falling asleep. However, this measure does not provide information regarding the degree of sleep fragmentation;
- $\left(\frac{1}{1+\frac{SFI2}{TST}}\right)$, accounts for the degree of sleep fragmentation by considering only transitions to stage N1 or awakenings.

The coefficients $w_{i}$ are the weights associated with each term of the SQI. In this study, a subject-specific calibration method is proposed to determine these weights, which are optimized for everyone via an iterative procedure that maximizes the Pearson correlation between the estimated SQI and subjective sleep quality score derived from the PSQI. This subject-specific optimization allows the model to account for inter-individual variability in sleep perception. As a result, the SQI serves as a personalized index, with the learned weights reflecting the individual’s perception and subjective experience of sleep quality. Its computation requires an initial calibration phase performed individually for each subject, based on multiple nights of recording and corresponding PSQI-derived sleep quality evaluations. Once calibrated, the SQI can be applied to subsequent recordings for the same subject. Further details regarding the optimization process are provided in Section 3.5.2. From the given formulation, it follows that SQI ∈ [0%, 100%], where 100% = optimal sleep and 0% = severely disturbed sleep (poor quality). A percentage scale for sleep classification based on the SQI value is shown in Table 4.

Although the proposed SQI relies on sleep parameters already established in the literature, its contribution lies in two main aspects. First, the SQI is designed to be directly integrated into the automatic sleep staging pipeline. Once sleep stages have been automatically classified, all parameters required for sleep quality assessment are extracted from the same processing workflow, enabling an objective, fully automated estimate of sleep quality without additional questionnaires or manual evaluations. Second, unlike existing indices that typically combine sleep parameters using fixed criteria, the proposed SQI incorporates a subject-specific adaptation mechanism. The contribution of each sleep parameter is normalized and weighted according to the individual’s sleep characteristics, allowing the index to account for inter-subject variability. This personalization aims to provide a more representative assessment of sleep quality at the individual level while preserving the objective nature of PSG-derived measurements.

#### 3.5.1. Description of the Dataset Used for Validating the SQI

To validate the proposed SQI’s ability to capture variations in sleep duration, continuity, and intensity, a dataset including sleep staging information and the parameters required for SQI computation (TST, TN3 or %N3, WASO, and SFI2), along with a sleep quality index, is needed. A dataset that meets these criteria is the “Sleep Architecture in Response to a Late Evening Competition in Team-Sport Athletes” dataset, collected by J. Vitale et al. at the IRCCS Istituto Ortopedico Galeazzi (Milan, Italy) [19]. The study investigated the impact of a late-evening competition on sleep patterns and recovery in team-sport athletes. The dataset includes 16 male competitive athletes (mean age 25.4 ± 1.4 years) monitored at home over four consecutive nights: two baseline nights before the competition (PRE2, PRE1), the night of the event (EM), and the following night (POST1). Sleep data were acquired using a self-applicable polysomnographic device (Sleep Profiler, Advanced Brain Monitoring), which records EEG, EOG, and electromyographic (EMG) signals and has been validated against standard polysomnography. Sleep staging was automatically performed in 30 s epochs using dedicated software. In addition, participants completed sleep diaries to provide complementary subjective information. Chronotype was assessed using the Horne–Ostberg Morningness–Eveningness questionnaire, baseline sleep quality using the PSQI, and perceived recovery using the Total Quality Recovery (TQR) scale. The dataset provides the following parameters:

- Bedtime, i.e., the time when the participant attempted to fall asleep;
- Wake-up time, i.e., the time of final awakening;
- TST, i.e., the time spent in bed minus the minutes spent awake;
- SE, i.e., the ratio of total sleep time to time spent in bed, expressed as a percentage;
- % REM sleep, i.e., valid REM sleep hours divided by total sleep hours;
- % Stage N1, i.e., valid Stage N1 sleep hours divided by total sleep hours;
- % Stage N2, i.e., valid Stage N2 sleep hours divided by total sleep hours;
- % Stage N3, i.e., valid Stage N3 sleep hours divided by total sleep hours;
- SOL, i.e., the interval between the attempt to sleep and the onset of the first three consecutive non-wake periods at the beginning of the night;
- WASO, i.e., the total minutes of wakefulness following the onset of sleep until the end of the recording;
- Cortical arousals, i.e., the number of cortical activation episodes per hour of sleep.

In addition, the sleep diary was used to record subjective rest quality, rated on a scale from 0 (very poor) to 10 (optimal).

#### 3.5.2. SQI Calculation and Weight Optimization

The SQI calculation was implemented using a MATLAB (ver. R2024b) script that loads the dataset “Sleep Architecture in Response to a Late Evening Competition in Team-Sport Athletes” (lines 5–20) and extracts the parameters needed to calculate the SQI for the 11 subjects and the 4 nights of observation. Subjects were selected based on the availability of all data necessary for the SQI calculation (e.g., number of nighttime awakenings). In fact, 4 subjects lacked the number of awakenings. This selection was performed as a preprocessing step before the SQI computation. Another subject was excluded because its sleep quality scores were constant, resulting in an undefined Pearson correlation coefficient.

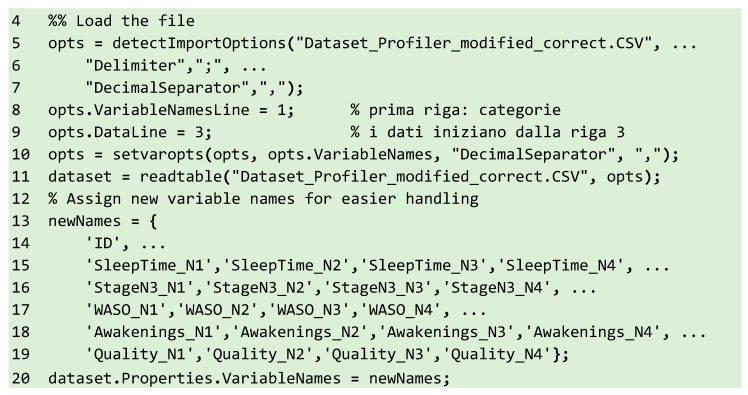



The proposed approach requires a customization phase to tune the weights used in the SQI calculation based on sleeper characteristics and habits/sensations. For each subject, a train/test strategy was employed to optimize the SQI’s weights ($w_{i}$) to maximize the Pearson correlation coefficient between the calculated SQI and the subjective SQI across three nights (Night 1–3). Afterward, the customized SQI was validated on the fourth sleep night by comparing the calculated SQI with the subjective SQI (Night 4). The Pearson correlation coefficient ($r_{p}$), which measures the linear relationship between two variables x and *y*, is defined as follows:

(9) $$r_{p}=\frac{\sum_{i=1}^{n}\left(x_{i}-\bar{x})(y_{i}-\bar{y}\right)}{\sqrt{\sum_{i=1}^{n}{\left(x_{i}-\bar{x}\right)}^{2}}\sqrt{\sum_{i=1}^{n}{\left(y_{i}-\bar{y}\right)}^{2}}}$$

where $x_{i}$ represents the SQI value, $\bar{x}$ its mean, $y_{i}$ the subjective sleep quality score, $\bar{y}$ its mean, and *n* is the number of observations (three nights). Although the number of observations per subject is limited (three nights), this approach is intended as a preliminary subject-specific calibration. A value of $r_{p}$ close to 1 indicates a strong positive linear correlation, whereas values close to 0 indicate no linear correlation. The correlation was computed using the MATLAB (ver. R2024b) function “corr” with the ‘Pearson’ option (line 77).

Specifically, the weights $w_{1}$, $w_{2}$, and $w_{3}$ were varied in steps of 0.01, while $w_{4}$ was calculated as $w_{4}=1-(w_{1}+w_{2}+w_{3})$. For each combination of weights, the script computes the SQI across three nights and evaluates the Pearson correlation between the SQI and each subject’s subjective sleep quality. The optimal set of weights maximizing this correlation is stored and reported for each subject (lines 26–84).

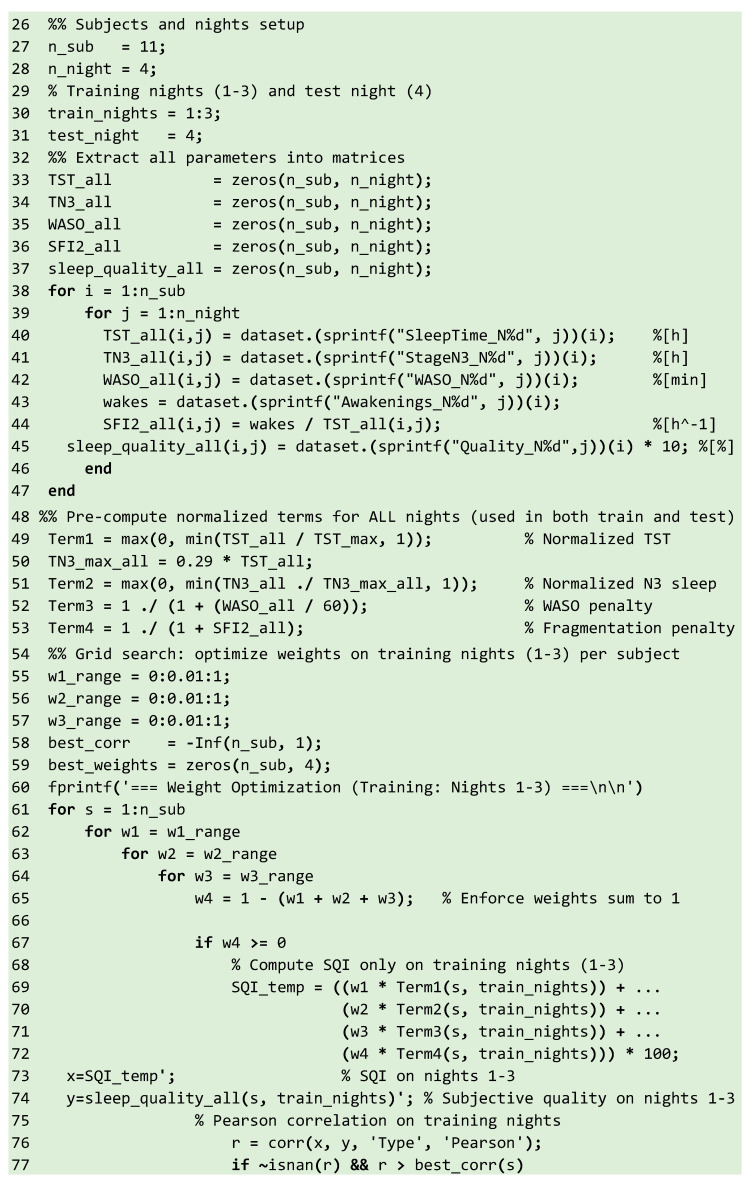

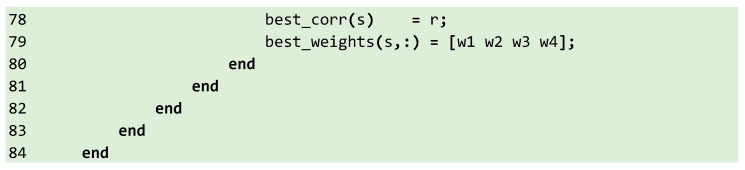





Finally, using the optimized weights, the script calculates the SQI on the fourth night (Night 4) and compares it with the corresponding subjective SQI, determining, for each subject, the absolute error to derive the mean absolute error for the subject cohort.

## 4. Results

This section presents the results obtained from the proposed algorithm for automatic sleep staging and sleep quality assessment. The performance of the two-stage deep learning model is first analyzed in terms of classification metrics, including accuracy, precision, recall, and F1-score, as well as confusion matrices, using the multimodal feature set adopted for training and testing. Afterward, the algorithm is validated on subjects not included in the training and testing phases, using the previously trained and validated classifier, to assess its generalization capability. Finally, the experimental validation of the proposed SQI is reported, demonstrating its capability to provide a comprehensive and objective evaluation of sleep quality by integrating multiple sleep domains, including duration, intensity, continuity, and fragmentation.

### 4.1. Performance of the Two-Stage DL Model for Sleep Staging

**Figure 8:**
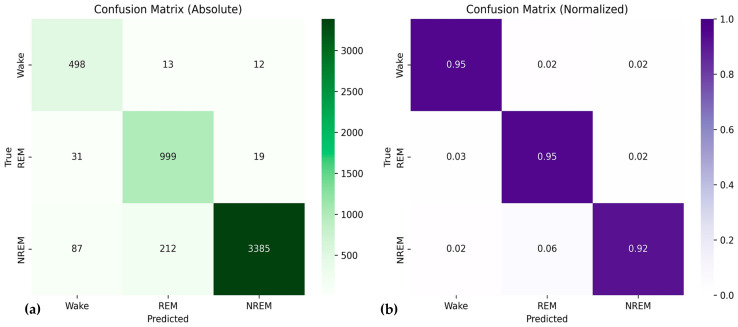
Absolute (**a**) and normalized (**b**) confusion matrices for the WRN classifier with the multimodal feature set.

After developing the WRN and NREM classifiers, both models were trained and evaluated using a multimodal feature set comprising 56 principal components (PCs) extracted from EEG signals, eight PCs from EOG, two respiratory-related features derived from the PPG signal, and eight cardiac-related features also derived from the PPG signal. The performance of the WRN classifier on the test set is illustrated in Figure 8 through both absolute (a) and normalized (b) confusion matrices, together with the corresponding evaluation metrics, including precision, recall, F1-score, and overall accuracy, which are summarized in Table 5. The WRN model achieves an overall accuracy of 92.9%.

**Figure 9:**
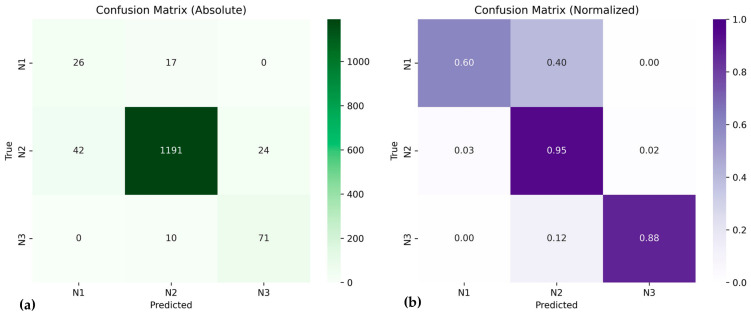
Absolute (**a**) and normalized (**b**) confusion matrices for the NREM classifier with the multimodal feature set.

Similarly, the performance of the three-class NREM classifier is reported in Figure 9, which presents both absolute (a) and normalized (b) confusion matrices. The associated evaluation metrics are summarized in Table 6. The NREM classifier attains an overall accuracy of 93.3%.

**Figure 10:**
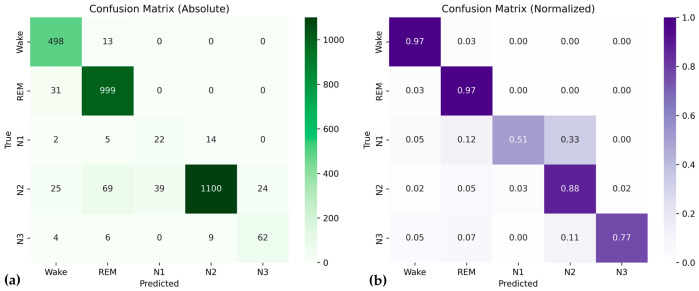
Absolute (**a**) and normalized (**b**) confusion matrices for the 5-class classifier with the multimodal feature set.

The two three-class models were then integrated in a cascaded architecture to obtain a five-class sleep staging system. The test set performance is shown in Figure 10, which includes both absolute (a) and normalized (b) confusion matrices, and the corresponding evaluation metrics are reported in Table 7.

The framework shows a lightweight memory footprint, with model sizes of 1.91 MB for the WRN classifier, 1.23 MB for the NREM classifier, and a total of 3.14 MB for the 5-class classifier. The model files are available in the Supplementary Materials (File S1).

To assess the potential impact of data augmentation on model generalization and to mitigate concerns regarding synthetic overfitting, statistical leakage, and artificial inflation, we performed a LOSO validation analysis. Unlike random-sample-based splitting strategies, LOSO ensures that all recordings from a given subject are excluded from training and used only for testing, thereby providing a more stringent evaluation of subject-independent generalization. This protocol is particularly relevant in sleep stage classification, where inter-subject variability may otherwise lead to overly optimistic performance estimates. Table 8 reports the mean and standard deviation of precision, recall, F1-score, and overall accuracy obtained using LOSO validation.

As reported in Table 8, the LOSO evaluation yielded an overall accuracy of 86.7% ± 3.1%, with weighted-average F1-score, precision, and recall values of 87.0% ± 3.2%, 88.8% ± 3.0%, and 86.2% ± 3.2%, respectively. Although the macro-average metrics were lower (F1-score = 56.3% ± 10.1%), reflecting the persistent difficulty of accurately classifying minority sleep stages (particularly N1 and N3), the overall performance remained consistent across training and testing. These results indicate that the model retains a reasonable ability to generalize to unseen subjects, reducing concerns that the reported performance is primarily driven by information leakage introduced during data augmentation.

To investigate whether more advanced class-balancing strategies could improve the classification of minority sleep stages, we compared the proposed data augmentation method with two widely used oversampling techniques, namely ADASYN (Adaptive Synthetic Sampling) and SMOTE (Synthetic Minority Over-Sampling Technique). All methods were evaluated under the same experimental conditions, and their performance (Table 9) was assessed using precision, recall, F1-score, and total accuracy.

As shown in Table 9, the proposed data augmentation strategy outperformed both ADASYN and SMOTE across all primary evaluation metrics, including total accuracy and weighted-average F1-score. Although the macro-average precision remained identical across the three methods, the proposed augmentation achieved higher macro-average recall and F1-score, indicating a better balance between majority and minority class performance. These results suggest that, for the dataset considered, the adopted augmentation strategy is more effective than other synthetic oversampling methods at mitigating class imbalance. Nevertheless, the classification of N1 and N3 stages remains challenging, highlighting an avenue for future research involving more sophisticated balancing strategies and architecture-level modifications.

To complete the characterization of the sleep staging model, we determined the inference time latency, throughput, CPU utilization, and estimated energy consumption. Latency was measured over 100 inference iterations (after 10 warm-up iterations) using a single-sample input, quantifying latency as mean inference time and percentiles (P95 and P99). Also, energy consumption was measured using a CPU utilization-based model, with instantaneous power consumption determined by scaling the CPU TDP (Thermal Design Power) of 15 W (Intel Core i5-1334U) based on CPU usage. Thus, the energy per inference was then calculated by multiplying the mean inference time by the estimated power. This process offers a means of comparing computing performance across several feature sets on the same hardware, albeit approximately. Table 10 reports the characterization of the two-stage sleep staging model.

The preprocessing time includes feature loading and sequence creation, which are performed before sleep staging; the model requires only 2.75 ms per sequence for inference. These results support the characterization of the proposed model as resource-efficient. Specifically, the inference latency is less than 0.01% of the 30 s sleep epoch, providing substantial computational headroom for real-time operation. Furthermore, the low CPU utilization (15.75%) and estimated energy consumption (7.01 mJ per inference) indicate that the model imposes a modest computational burden and can operate without requiring dedicated high-performance computing resources. Although benchmarking was performed on a general-purpose CPU rather than a dedicated embedded platform, the combination of low latency, high throughput, and limited resource utilization suggests that the proposed framework is suitable for deployment on resource-limited hardware.

To assess the contribution of each biosignal modality to the overall performance of the proposed multimodal framework, an ablation study was conducted [53,54]. To investigate the contribution of the additional biosignal modalities, an incremental modality analysis was performed. Starting from the EEG-only configuration, the model was retrained by progressively incorporating EOG and PPG signals while maintaining the same architecture, training protocol, and evaluation procedure. The resulting performance was then compared with that of the complete multimodal model. This analysis was designed to evaluate the extent to which each additional modality contributes to sleep-stage classification performance and to assess the robustness of the proposed framework when specific signal sources are unavailable. Table 11 reports the five-class classification performance metrics and model size for each feature set, allowing the contribution of the additional EOG and PPG modalities to be assessed relative to the EEG-only baseline.

### 4.2. Validation of the Staging Algorithm on Subjects External to the Training/Testing Dataset

Once the sleep staging algorithm was developed, as described in Section 3.4, it was tested on several subjects not included in the dataset used for training and testing the model, to verify its performance when applied to data not previously considered. Therefore, 14 subjects were selected from the BOAS dataset who had not been selected during the previous subject selection step performed during the construction phase of the dataset used for training and testing the sleep staging model. The only requirement considered in subject selection was the presence of the same set of biosignals (EEG, EOG, and PPG) as those used to construct the training/testing dataset. Subsequently, the signals were manually inspected to remove uniformly noise- and artifact-corrupted portions, staging them both before and after removal to verify their impact on the classification algorithm’s overall performance. In addition, each signal was tested with two values of the “max_gap” parameter (5 and 25), used to smooth spurious transitions in REM sleep, enabling the evaluation of the smoothing effect on the model’s predictions and the overall performance of the sleep staging algorithm. The performance of the classification algorithm is quantified using *accuracy* as the evaluation metric, defined as:

(10) $$Accuracy=\frac{n{}^{\circ}\;of\;correctly\;predicted\;sequences}{n{}^{\circ}\;of\;total\;sequences\;of\;the\;subject}$$

Table 12 shows the accuracy values of the staging algorithm for an entire night’s sleep, compared with the staging reported in the dataset’s annotation file, determined by consensus among three medical experts. In detail, Table 12 reports the accuracy obtained by the pipeline pre- and post-application of the heuristic rules (discarding W↔N3 direct transitions and REM smoothing) and with them applied to both the full signals and signals after removing portions affected by artifacts. In this way, the impact of the heuristic rules on the pipeline performance is established. In addition, Table 12 reports the accuracy values for two values of the “max_gap” parameter: 5 and 25. To provide a comprehensive quantitative validation of the proposed architecture’s impact, a comparative analysis was performed against a probabilistic baseline using the HMM approach. Therefore, in Table 12, the HMM approach was evaluated under identical conditions using both raw and artifact-cleaned signals.

**Figure 11:**
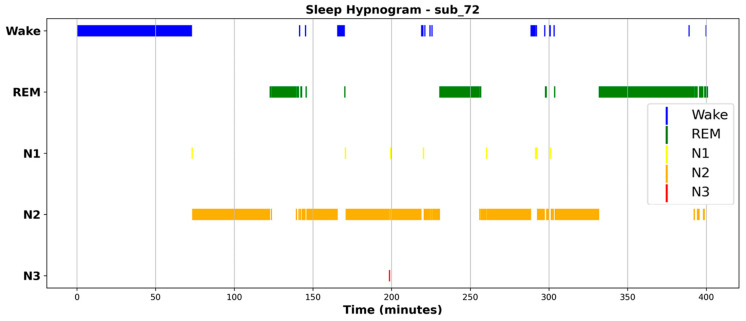
An example of a hypnogram generated by the developed sleep staging algorithm for subject 72 (with no heuristic rules), who achieved the highest accuracy score among all subjects.

**Figure 12:**
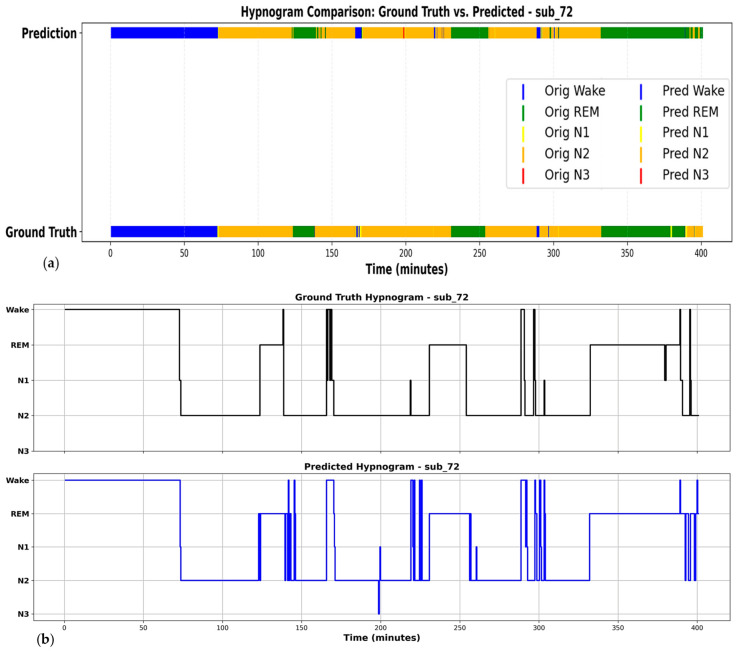
Comparison between the hypnograms generated by the automatic sleep staging algorithm (namely, the predicted hypnogram) and the ground truth represented by the sleep staging data from dataset for subject 72, for both representation modes: (**a**) single-layer and (**b**) multilayer hypnogram.

Figure 11 shows the hypnogram for subject 72, the subject for whom the model achieved the best performance (accuracy = 91.99%). Furthermore, Figure 12 compares the hypnogram obtained using the staging algorithm with the ground truth, represented by the staging provided by the BOAS dataset.

**Figure 13:**
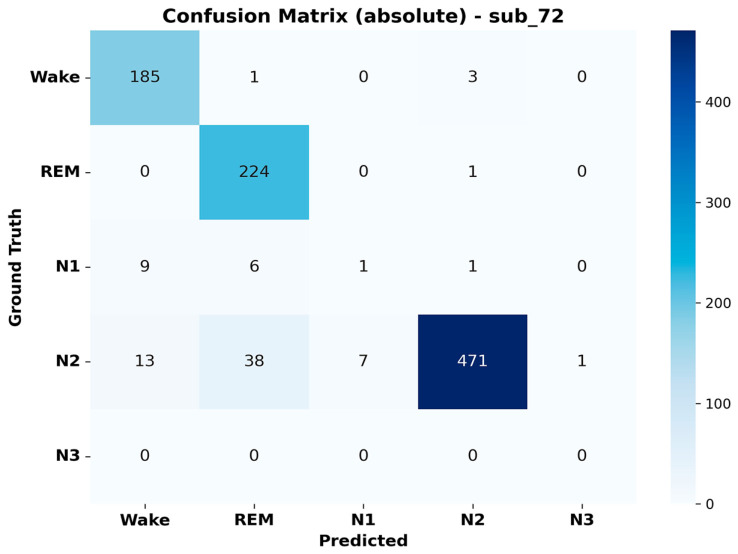
Confusion matrix of the predictions (in terms of sequences) provided by the sleep staging algorithm with the ground truth represented by the annotations in the BOAS dataset for subject 72.

Figure 13 shows the confusion matrix of the staging model’s predictions for sequences covering the entire night for subject 72, using the manual sleep scoring from the BOAS dataset as the ground truth.

#### Validation of the Staging Algorithm on Subjects of the ISRUC Datasets

To emphasize the algorithm’s performance and to demonstrate that there is no risk of synthetic overfitting, statistical leakage, and artificial inflation of performance, we carried out cross-dataset validation on a public sleep database, the ISRUC-Sleep dataset [55], to thoroughly assess the generalization capacity of our suggested two-stage LSTM framework beyond the BOAS dataset. This dataset was chosen because it provides a set of signals compatible with our complete feature set (cardiorespiratory, EEG, and EOG features). As the dataset does not provide the PPG signal, cardiorespiratory features were extracted from the ECG signal. The ISRUC-Sleep dataset is divided into three subgroups: Subgroup I (SG-I) comprises 100 subjects with signs of sleep disorders (sleep apnea); Subgroup II (SG-II) comprises data from 8 subjects with signs of sleep disorders (sleep apnea) collected across two acquisition sessions; and Subgroup III (SG-III) comprises the control group, comprising data from 10 healthy subjects. A set of 25 randomly selected subjects from SG-I of the ISRUC dataset were used to test the model. Using the same preprocessing and feature-extraction pipeline outlined in Section 3.4, we used a zero-shot evaluation technique in which the model was assessed directly on unseen target datasets. In this way, the model’s efficacy in subjects with sleep disorders was verified. Table 13 summarizes the performance (mean ± std) of the proposed automatic sleep staging classifier across the ISRUC (SG-I) dataset.

Specifically, the automatic sleep staging algorithm achieved a mean accuracy of 78.1%. Due to variations in recording hardware, sampling rates, electrode placement, and population characteristics and conditions (i.e., healthy and pathologic) between the BOAS and ISRUC datasets, the performance degradation in accuracy (−12.7%) relative to the BOAS test set is similar to that observed when state-of-the-art models are assessed on an external dataset. Specifically, well-known sleep staging models like TinySleepNet and XSleepNet2 demonstrated a notable decline in performance during cross-dataset testing compared to that during validation [56,57].

### 4.3. Experimental Validation of the Proposed Sleep Quality Index

The optimal subject-specific weights, resulting from the calibration procedure described in Section 3.5.2, are summarized in Table 14 and used to compute the SQI and the correlation from the optimization process. As previously described, after the weight optimization carried out on the first three nights, the SQI was validated on the fourth sleep night for each subject using the optimized weights; Table 15 compares the calculated SQI and the subjective SQI for each subject resulting from the validation (Night 4), reporting the corresponding absolute error.

The validation results demonstrate that the proposed calibration procedure can effectively adapt the SQI to individual sleep characteristics. In the independent fourth-night validation, the calculated SQI had a mean absolute error of 10.81 points relative to the subjective SQI. The median absolute error on the Night-4 validation is 11, which is close to the MAE (10.8 points), suggesting a relatively symmetric error distribution with limited skewness and no substantial influence from extreme outliers. Although several subjects exhibited close agreement between objective and subjective scores, larger errors observed in some individuals indicate that further investigation on larger cohorts is necessary to assess the robustness and generalizability of the proposed personalization strategy.”

## 5. Discussion

This section analyzes and interprets the results presented in Section 4, with a particular focus on the performance of the automatic sleep classifier and the validation of the automatic staging algorithm (Section 5.1). Section 5.2 discusses the results regarding the SQI obtained by optimizing the weights for each subject.

### 5.1. Performance Evaluation of the Automatic Sleep Classifier and Validation of Staging Algorithm

A multimodal setup comprising 56 EEG PCs, eight EOG PCs, two respiratory, and eight cardiac PPG features was used to assess the automatic sleep classifier. With this feature set, the three-class WRN classifier achieved a 92.9% overall accuracy and a macro-average F1-score of 90.2% (Table 5). Wake epochs were identified with an F1-score of 87.4%, precision of 80.8%, and recall of 95.2%, indicating good separability of this state. REM sleep classification benefited from the inclusion of EOG-related PCs capturing rapid eye movements, yielding an F1-score of 87.9%, precision of 81.6%, and recall of 95.2%. NREM sleep showed the most consistent performance (F1-score of 95.4%, precision of 99.1%, recall of 91.9%), confirming robust classification. The WRN classifier has a memory occupation of just 1.91 MB.

Using the same multimodal feature set, the three-class NREM classifier achieved an overall accuracy of 93.3% and a macro-average F1-score of 74.6% for classifying N1, N2, and N3 stages, as reported in Table 6. The N1 stage exhibited the lowest performance, with an F1-score of 46.8%, a precision of 38.2%, and a recall of 60.5%, reflecting the difficulty in correctly identifying this transitional sleep stage. In contrast, the N2 stage showed consistently high performance, with an F1-score of 96.2%, precision of 97.8%, and recall of 94.7%, indicating a robust and reliable classification. The N3 stage also showed good detection of deep sleep periods, achieving an F1-score of 80.7%, precision of 74.7%, and recall of 87.7%, indicating a slight tendency to over-predict the N3 class. Overall, the multimodal feature set enables effective discrimination among NREM sub-stages, with particularly strong performance for N2 and N3, while N1 is the most challenging class to identify. This behavior is justified by the underrepresentation of N1 and N3 sequences in the training and test datasets, as discussed in Section 3.3, which reduces the classifier’s exposure to these stages and consequently limits its ability to generalize these classes effectively. The NREM classifier shows a lightweight memory footprint with a model size of 1.23 MB.

Finally, the cascaded five-class sleep staging classifier, implemented under the same multimodal configuration, reached an overall accuracy of 90.8% and a macro-average F1-score of 78.9%, as reported in Table 7, supporting the reliability of the full architecture. The Wake stage was identified with high reliability, achieving an F1-score of 92.0%, precision of 88.9%, and recall of 95.2%. Similarly, REM sleep showed strong performance, with an F1-score of 93.3%, precision of 91.5%, and recall of 95.2%, indicating a limited number of misclassifications. Within the NREM sub-stages, N1 remained the most difficult class to detect, with an F1-score of 42.3%, precision of 36.1%, and recall of 51.2%, due to its transitional nature and overlap with adjacent stages such as Wake and N2, but mainly to the under-representation of the N1 class in the training/test dataset. In contrast, N2, which accounted for the largest proportion of total sleep time, showed consistent performance, yielding an F1-score of 92.4%, precision of 98.0%, and recall of 87.5%. N3 achieved an F1-score of 74.3% with a precision of 72.1% and recall of 76.5%, confirming effective detection of deep sleep, though with a tendency toward overestimation, as indicated by the high recall. The complete cascaded architecture has an overall model size of 3.14 MB. The performance across sleep stages suggests that, despite data augmentation to compensate for class imbalance, the less represented classes (N1 and N3) perform worse than the majority classes, as indicated by the macro-average metrics reported in Table 7.

Furthermore, to quantify the contribution of each biosignal modality, we conducted an ablation study (Table 11) by systematically training and evaluating the model across four different feature sets (EEG-only, EEG + EOG, EEG + PPG, and EEG + EOG + PPG). This experimental design allows us to assess the impact of each signal on the overall performance. The results show that EEG-only provides strong performance (89.9% accuracy), confirming it as the dominant source of discriminative information for sleep stage classification. The inclusion of EOG yields modest improvements in accuracy (+0.8%, from 89.9% to 90.7%), indicating that ocular activity provides complementary information, particularly during transitional stages such as REM. In contrast, adding PPG provides only a limited additional benefit, suggesting a partially informative contribution, obtaining an accuracy of 90.0% (+0.1% with respect to the only-EEG feature set). The complete multimodal feature set (EEG + EOG + PPG) achieves the best overall performance (90.8% accuracy, +0.9% over EEG-only). A similar trend is observed in model size: the EEG-only model is 2.95 MB; EEG + EOG increases slightly to 3.04 MB (+0.09 MB); EEG + PPG increases to 3.06 MB (+0.11 MB); and the full multimodal model reaches 3.14 MB (+0.19 MB compared to EEG-only). The limited performance and model size variations across feature sets suggest that the framework is primarily EEG-driven, with EOG providing the most consistent complementary contribution. At the same time, PPG has a relatively small impact on performance. These results suggest a limited sensitivity to the removal of individual modalities, with performance remaining largely stable when a single signal is excluded.

While the previous results highlight the effectiveness of the proposed models under training and testing conditions, an additional validation phase was conducted to assess their generalization performance on subjects outside the training/test dataset, as would happen in a real-world scenario. The sleep staging algorithm achieved accuracy ranging from 80.26% to 91.99% across the 14 selected subjects outside the training/test dataset. Removing signal portions affected by artifacts typically improves model performance by eliminating sections that mislead the model, thereby enhancing the algorithm’s decision-making capabilities, as is reported in Table 12. However, for some subjects (e.g., 52, 58, 62, and 96), signal cleaning led to a slight degradation of the sleep staging algorithm’s performance, an effect attributable to the removal of signal portions that the algorithm was still able to classify correctly, despite the presence of artifacts in one or more of the analyzed biosignals. However, the phase-sequence interruption caused by the removal operation likely led to some staging errors in those signal portions. Also, the effect of the heuristic rules on the pipeline performance was evaluated, comparing the accuracy pre- and post-their application; in most cases, the use of moderate smoothing (max_gap = 5) on epochs classified as REM has a beneficial effect on model performance, leading to an increase, even a significant one (e.g., for subjects 58, 61, 80, 81 and 96), in accuracy. However, a more aggressive smoothing, with a max_gap parameter of 25, tends to introduce errors during transition phases involving passage through REM sequences, thereby reducing the model’s performance across almost all subjects.

Furthermore, Table 12 compares our heuristic rules with the HMM approach; the test results show that across the cohort of unseen subjects, the HMM approach generally outperforms the LSTM model regularized with heuristic rules. The behavior is primarily due to the HMM’s inherent strength in capturing strictly probabilistic transition dependencies between subsequent epochs over long temporal chains, allowing it to naturally smooth out sudden, non-physiological phase transitions without relying on a rigid threshold-based constraint. For instance, under the cleaned signal condition, the HMM achieves 91.99% accuracy for subject 72, compared with 90.74% obtained with our heuristic rules. This result suggests that integrating a probabilistic transition matrix into the post-processing pipeline or exploring hybrid deep-probabilistic models represents a promising direction for further enhancing the reliability of automatic sleep staging.

When comparing the hypnogram obtained from the sleep staging algorithm with the reference one, there is excellent agreement between the predictions from the sleep staging model and ground truth (Figure 12). The model faithfully replicates the macro-architecture of sleep, accurately reflecting the duration and sequence of NREM-REM cycles, with only minor discrepancies in phase transitions. Figure 13 shows the confusion matrix of the staging model’s predictions over the entire sleep night, using the BOAS dataset’s sleep scoring as the ground truth. The distribution of values highlights a good overall accuracy, with most sequences correctly classified along the main diagonal. The best performance is observed for the REM and N2 stages, with a high number of correct classifications. The Wake state is also well identified, although with some errors towards N2. As previously discussed, the N1 stage appears to be the most challenging, since most of them are confused with Wake and N2. Regarding the N3 class, good detection capability is observed, though some misclassifications toward N2 indicate an overlap between the two stages.

Tests conducted on unseen subjects demonstrated discrete variability in the algorithm’s performance across the subject cohort, with values slightly lower but still in line with those obtained in the model test (90.8%), reflecting the natural variability in signals across subjects. In addition to unseen subject validation from the BOAS dataset (Section 4.2), a cross-dataset validation using the ISRUC dataset was performed (Section Validation of the Staging Algorithm on Subjects of the ISRUC Datasets). Both validations were essential for assessing the model’s ability to handle unseen data.

### 5.2. SQI Validation and Correlation with the Subjective Sleep Quality Index

Before discussing the performance and optimization of the proposed SQI, it is worth evaluating the suitability of the validation dataset, which considers conditions of shift in the sleep–wake rhythm. Specifically, after evening matches, a significant delay in bedtime was observed (on average, approximately 2 h and 29 min compared to pre-game nights) with a corresponding delay in wake time (on average, approximately 9 h and 20 min). Despite these temporal changes, the key sleep quality metrics considered within the SQI framework, including TST, SE, sleep stage distribution, SOL, WASO, and arousal index, did not show substantial variations between the different conditions. Similarly, perceived recovery (TQR) scores and subjective sleep quality remained essentially unchanged [19]. These findings suggest that changes in sleep timing alone do not necessarily imply alterations in intrinsic sleep quality, as captured by the proposed index, emphasizing the importance of multiple-night recordings for distinguishing circadian rhythm effects from true changes in sleep quality. These results also demonstrate the adequacy of the selected dataset for validating the proposed SQI, enabling analysis across multiple consecutive nights for each subject.

From the calibration process aimed at maximizing the Pearson correlation coefficient for each subject (Table 14), it is evident that the weights ($w_{i},\;i=1,\;2,\;3,\;4$) differ significantly across the 11 subjects considered. This lack of correlation in weight across subjects is attributable to the purely subjective nature of sleep quality assessment, which is influenced by each subject’s needs, sensations, and habits. This result justifies the proposed approach of customizing the SQI for each tested subject by analyzing metrics derived from sleep scoring and subjective sleep assessments over multiple consecutive nights to optimize the SQI weights ($w_{i}$) for each subject. Once these weights have been determined, the customized SQI will be used to quantify sleep quality over subsequent nights objectively.

The calibration results in Table 14 demonstrate a successful weight optimization process across the initial training nights (Nights 1–3). Despite the restricted calibration window, a robust linear relationship was achieved between the optimized SQI and subjective scores for most participants. Independent testing on Night 4 confirms this performance, with the calculated SQI yielding an MAE of 10.81 points against subjective sleep quality profiles (Table 15). The median absolute error of 11.00 points closely matches the MAE, indicating a symmetric error distribution unaffected by severe outliers. Although most individuals showed strong concordance, the wider error margins in some cases emphasize the need to validate this subject-specific strategy in larger cohorts to assess its broader generalizability (Table 15).

In this context, weight optimization must be intended as an initial calibration phase for the system. In practice, before the index can be used operationally, a limited number of nights of sleep must be collected for each subject, during which both sleep-scoring data and subjective assessments are collected through sleep diaries and the PSQI questionnaire. The latter enables the estimation of a subjective sleep quality score, which serves as a reference for optimizing the index weights. Once this phase is complete, the SQI can be used to provide an objective, personalized assessment of sleep quality on subsequent nights, without the need for further subjective input. The differences observed in some subjects, such as Subject 7, can be attributed to both inter-individual variability in sleep perception. The differences observed between the SQI and subjective sleep assessment highlight that sleep quality is a complex concept, influenced not only by objective parameters but also by subjective factors that are difficult to model.

Overall, the results indicate that the proposed SQI is a promising tool for quantitative assessment of sleep quality, with potential applications in personalized monitoring and integration into wearable systems for real-world sleep analysis.

## 6. Conclusions

This study presents the development of a two-stage sleep staging classifier, trained and tested on an optimized multimodal feature set including PCs/features of EEG, EOG, and PPG signals. These data were extracted from 25 subjects from the BOAS dataset. The test results demonstrated that the five-class classifier achieved high classification performance (90.8% total accuracy; Table 7) while maintaining a lightweight memory footprint (3.14 MB total memory usage), making it suitable for real-world applications. The model’s robustness was established through LOSO cross-validation and an independent cross-dataset evaluation using pathological records from the ISRUC-Sleep database. The stable performance maintained across this distinct cohort demonstrates that our feature optimization and cascading neural network maintain high diagnostic reliability when exposed to unseen clinical populations. Then, the classifier was integrated into an automatic sleep staging algorithm, which was validated on subjects from the BOAS dataset who were not involved in the training or testing phases. Using heuristic post-processing rules, the automatic sleep staging algorithm achieved accuracies ranging from 80.26% to 91.99% across the 14 selected subjects excluded from the training and testing datasets. In addition, the same validation was performed using an HMM-based approach on the same subjects, yielding improved performance and reaching a peak accuracy of 91.99%.

Finally, an SQI based on metrics derived from sleep staging was proposed, combining different sleep domains (duration, intensity, continuity-fragmentation) and overcoming the limitations of methods reported in the literature that consider only one sleep aspect. The SQI provides an objective, subject-specific assessment of sleep quality by requiring a calibration phase in which its weights are adapted to the individual’s needs, sensations, and habits. The proposed SQI was validated on an independent dataset containing data from 11 subjects across 4 consecutive sleep nights. A train/test approach was followed, optimizing weights on three nights (Nights 1–3) and testing the SQI on an independent night (Night 4). These findings confirm the effectiveness of the proposed calibration strategy and support the potential of the SQI for objective, personalized, and fully automated sleep quality monitoring. Despite promising results, the main limitation is the small number of calibration (n = 3) and validation (single night) nights per subject due to the scarcity of datasets combining sleep staging-derived parameters with subjective sleep quality scores. A larger validation dataset is required to confirm its robustness and generalizability across diverse populations and sleep patterns, which will be addressed in future work. Also, the next step is to integrate the proposed sleep staging algorithm into dedicated software for automatic sleep scoring, analyzing biosignals acquired from the sleep mask. In addition, while the current version operates in offline mode using the full-night feature set, the algorithm can be adapted for online sleep staging during signal acquisition, enabling continuous, incremental predictions throughout recording. Furthermore, the SQI computation module is intended to be integrated into the same software environment as the automatic sleep staging system. In this way, once sleep staging is complete, the algorithm can automatically extract the required parameters (TST, TN3, WASO, SFI2) and compute the corresponding SQI, enabling a fully automated pipeline for both sleep classification and quality assessment.

## Figures and Tables

**Table 1:** A standardized set of sleep-scoring data reported in the AASM manual includes parameters useful for assessing sleep quality.

N.	Parameter	Description
1	Lights out clock time (h:min)	Hour of “lights out”
2	Lights on clock time (h:min)	Hour of “lights on”
3	Total sleep time (TST, min)	Sum of all sleep epochs
4	Total recording time (TRT, min)	Interval between “lights out” and “lights on”
5	Sleep latency (SL, min)	Time between lights out and the first sleep period
6	Stage R latency (min)	Time between the first sleep period and the first REM period
7	Wake after sleep onset (WASO, min)	TRT-SL-TST
8	Percent sleep efficiency (%)	(TST/TRT) × 100
9	Time in each stage (min)	W, R, N1, N2, N3
10	Percent of TST in each stage (%)	(Time of each stage/TST) × 100

**Table 3:** Comparison of sleep quality indices and their parameters, as discussed in Section 2.2.

Reference	Sleep Quality Index	Parameters Used
Balakrishnan et al. [39]	$Sleep\;Index=\frac{\sum_{i=0}^{n}T_{i}\cdot W_{i}}{T_{o}\cdot NSS}$	$T_{i}$ (time spent in each sleep stage), $W_{i}\;$(corresponding weights), $T_{o}$ (total sleep time), and NSS (number of stage shifts)
Yoo et al. [40]	Ru−SATED score=R+S+A+T+E+D	Regularity (R), Satisfaction (S), Alertness (A), Timing (T), Efficiency (E), and Duration (D)
Nam et al. [41]	$Sleep\;Quality=\frac{TNR}{TST}\alpha +\left(100-N_{AE}\right)\beta +P_{T}\cdot \gamma$	NREM sleep duration (TNR), TST, number of apnea episodes (NAE), and total time spent in the primary sleep posture (PT); α, β, and γ are empirically determined weighting factors, equal to 0.5, 0.3, and 0.2
Shambroom et al. [42]	$ZQ=\left(TST\times 1+DST\times 1.5+RST\times 0.5\right)\;\;\;\;\;\;\;\;\;\;\;\;\;\;\;\;\;\;\;\;\;\;\;\;\;\;\;\;\;-(TIW\times 0.5+\frac{ATS}{15})\times 8.5$	DST is the duration of deep sleep, RST is the duration of REM sleep, TIW is the total time awake during the night, and ATS is the number of awakenings
Cheng et al. [43]	${SQ}_{obj}=(TST\times {\left(\frac{a_{m}}{a_{p}}\right)}^{2}+DST\times {\left(\frac{a_{m}}{a_{p}}\right)}^{2}\times 1.5\;\;\;\;\;\;\;\;\;\;\;\;\;\;\;\;\;\;\;\;\;\;\;\;\;\;\;\;\;+RST\times {\left(\frac{a_{m}}{a_{p}}\right)}^{2}\times 0.5\;\;\;\;\;\;\;\;\;\;\;\;\;\;\;\;\;\;\;\;\;\;\;\;\;\;\;\;\;-TIW\times {\left(\frac{a_{m}}{a_{p}}\right)}^{2}\times 0.5+\frac{ATS}{a_{A}})\times a_{p}$	a_m_ is the average optimal sleep duration, a_p_ is the personalized optimal sleep duration, and a_A_ represents a personalized normalization factor for awakenings
Miwa et al. [44]	$SQS=\frac{S_{D}}{S}$	Time in deep sleep (S_D_), total sleep period (S)
Han et al. [45]	$S_{Quality}=A\times W$	A represents the normalized magnitude of the acceleration vector, and W is a weight factor empirically assigned (e.g., W_supine_ = 0, W_prone_ = 1, W_left and right_ = 0.5)

**Table 4:** Percentage scale for classifying sleep quality based on the SQI score.

SQI	Sleep Quality
100–80%	Excellent
79–60%	Good
59–40%	Discrete
<40%	Poor

**Table 5:** Evaluation metrics of the WRN classifier trained and tested on the multimodal feature set.

	Precision [%]	Recall [%]	F1-Score [%]	Support for Each Class
Wake	80.8	95.2	87.4	523
REM	81.6	95.2	87.9	1049
NREM	99.1	91.9	95.4	3684
Macro avg	87.2	94.1	90.2	5256 (total support)
Weighted avg	93.8	92.9	93.1	5256 (total support)
Total accuracy	92.9%			5256 (total support)

**Table 6:** Evaluation metrics of the NREM classifier trained and tested with the multimodal feature set.

	Precision [%]	Recall [%]	F1-Score [%]	Support for Each Class
N1	38.2	60.5	46.8	43
N2	97.8	94.7	96.2	1257
N3	74.7	87.7	80.7	81
Macro avg	70.3	81.0	74.6	1381 (total support)
Weighted avg	94.6	93.3	93.8	1381 (total support)
Total accuracy	93.3%			1381 (total support)

**Table 7:** Evaluation metrics of the 5-class classifier trained and tested on the multimodal feature set.

	Precision [%]	Recall [%]	F1-Score [%]	Support for Each Class
Wake	88.9	95.2	92.0	523
REM	91.5	95.2	93.3	1049
N1	36.1	51.2	42.3	43
N2	98.0	87.5	92.4	1257
N3	72.1	76.5	74.3	81
Macro avg	77.3	81.1	78.9	2953 (total support)
Weighted avg	92.4	90.8	91.4	2953 (total support)
Total accuracy	90.8%			2953 (total support)

**Table 8:** Mean and standard deviation of precision, recall, F1-score, and overall accuracy obtained using the LOSO validation.

	Precision [%]	Recall [%]	F1-Score [%]
Macro avg	55.3 ± 8.7	60.4 ± 10.0	56.3 ± 10.1
Weighted avg	88.8 ± 3.0	86.2 ± 3.2	87.0 ± 3.2
Total accuracy	86.7% ± 3.1%		

**Table 9:** Performance comparison of the proposed data augmentation approach and alternative oversampling techniques (ADASYN and SMOTE) for addressing class imbalance in sleep stage classification.

Oversampling Techniques		Precision [%]	Recall [%]	F1-Score [%]
Proposed Data Augmentation	Macro avg	77.3	81.1	78.9
	Weighted avg	92.4	90.8	91.4
	Total accuracy	90.8%		
ADASYN	Macro avg	77.3	78.5	77.7
	Weighted avg	90.7	89.8	90.0
	Total accuracy	89.8%		
SMOTE	Macro avg	77.3	77.8	77.4
	Weighted avg	90.5	89.6	89.7
	Total accuracy	89.6%		

**Table 10:** Benchmarking results of the 5-class sleep staging model: inference time, latency, and energy consumption analysis.

Parameter	Value
Preprocessing time [ms]	6670.23
Mean latency [ms]	2.75
P95 [ms]	3.87
P99 [ms]	4.25
Throughput [sequences/s]	363.63
CPU Usage [%]	15.75
Estimated Energy [mJ]	7.01

**Table 11:** The 5-class classification performance metrics and model size across each feature set.

Feature Set		Precision [%]	Recall [%]	F1-Score [%]	Model Size (MB)
Only EEG	Macro avg	76.0	81.9	78.4	2.95
	Weighted avg	92.1	89.9	90.7	
	Total accuracy	89.9%			
EEG + EOG	Macro avg	78.2	80.7	79.3	3.04
	Weighted avg	92.4	90.7	91.4	
	Total accuracy	90.7%			
EEG + PPG	Macro avg	75.7	79.9	77.5	3.06
	Weighted avg	91.8	90.0	90.7	
	Total accuracy	90.0%			
EEG + EOG + PPG	Macro avg	77.3	81.1	78.9	3.14
	Weighted avg	92.4	90.8	91.4	
	Total accuracy	90.8%			

**Table 12:** Accuracy of the automatic staging algorithm, evaluated on 14 subjects not included in the training/testing dataset, pre- and post-application of the heuristic rules (two different “max_gap” parameters: 5 and 25) and HMM, and for different input configurations (integer signals and cleaned signals). The test with the lowest accuracy is highlighted in orange; the one with the highest accuracy is indicated in green.

Subject	Accuracy (Raw-Integer Signal)	Accuracy (Heuristic Rules-Integer)	Accuracy HMM (Integer Signal)	Accuracy (Raw-Cleaned Signal)	Accuracy (Heuristic Rules-Cleaned)	Accuracy HMM (Cleaned Signal)
52	86.23%	85.67% ^a^	88.84%	85.29%	83.86% ^a^	88.46%
		83.16% ^b^			81.31% ^b^	
58	84.49%	84.57% ^a^	88.76%	84.38%	84.46% ^a^	89.08%
		81.87% ^b^			76.75% ^b^	
60	84.13%	84.39% ^a^	87.10%	84.16%	83.79% ^a^	87.11%
		81.26% ^b^			80.94% ^b^	
61	85.38%	86.68% ^a^	88.81%	87.08%	88.14% ^a^	91.03%
		84.00% ^b^			87.17% ^b^	
62	90.32%	89.13% ^a^	91.34%	89.02%	88.42% ^a^	91.17%
		88.12% ^b^			87.39% ^b^	
72	87.51%	86.20% ^a^	91.51%	91.68%	90.74% ^a^	91.99%
		82.83% ^b^			89.39% ^b^	
74	82.61%	81.92% ^a^	84.15%	83.64%	82.63% ^a^	85.27%
		80.89% ^b^			81.48% ^b^	
78	81.32%	79.94% ^a^	84.09%	82.67%	80.08% ^a^	85.18%
		74.02% ^b^			77.10% ^b^	
80	82.32%	85.08% ^a^	83.27%	83.19%	86.28% ^a^	84.44%
		87.07% ^b^			88.31% ^b^	
81	82.35%	83.53% ^a^	84.41%	82.43%	83.53% ^a^	84.84%
		77.75% ^b^			76.31% ^b^	
82	83.44%	83.07% ^a^	85.51%	84.06%	84.06% ^a^	85.77%
		80.62% ^b^			81.21% ^b^	
84	84.01%	82.87% ^a^	84.75%	84.74%	84.45% ^a^	84.65%
		78.47% ^b^			84.06% ^b^	
96	80.26%	85.17% ^a^	81.55%	79.88%	83.83% ^a^	80.67%
		88.79% ^b^			87.61% ^b^	
101	85.05%	84.74% ^a^	88.16%	87.36%	86.82% ^a^	89.43%
		84.22% ^b^			83.66% ^b^	

^a^ max_gap = 5; ^b^ max_gap = 25.

**Table 13:** Validation results (mean ± std) of the sleep stage classifier on the ISRUC(SG-I) dataset.

Accuracy [%]	Precision [%]	Recall [%]	F1-Score [%]
78.1 ± 5.5	80.3 ± 4.8	77.5 ± 3.9	76.2 ± 4.2

**Table 14:** Weights optimized for each subject on the three sleep nights (Night 1–3), derived by the Pearson coefficient’s optimization.

ID	$w_{1}$	$w_{2}$	$w_{3}$	$w_{4}$	Optimized Correlation
1	0.11	0.10	0.11	0.68	0.66
2	0.27	0.11	0.16	0.46	0.98
3	0.11	0.20	0.53	0.16	0.99
4	0.20	0.15	0.31	0.34	0.99
5	0.24	0.42	0.14	0.20	0.99
6	0.14	0.19	0.54	0.13	0.99
7	0.69	0.10	0.11	0.10	0.70
9	0.11	0.68	0.11	0.10	0.48
10	0.11	0.25	0.20	0.44	0.99
11	0.11	0.10	0.11	0.68	0.33
12	0.53	0.12	0.20	0.15	0.99

**Table 15:** Comparison between the estimated SQI (SQI Night 4 columns) and the corresponding sleep quality scores on the Night 4 (Subj-SQI Night 4 columns).

ID	SQI Night 4	Subj-SQI Night 4	Absolute Error
1	63	50	13
2	79	80	1
3	84	80	4
4	84	90	6
5	59	70	11
6	87	80	7
7	72	50	22
9	61	80	19
10	83	60	23
11	92	90	2
12	79	90	11

## Data Availability

The data are available upon request.

## Supplementary Materials

The following supporting information can be downloaded at: https://www.mdpi.com/article/10.3390/s26134091/s1, File S1: saved_models_working_complete_feature_set.zip; File S2: Sleep Staging Pipeline.py.

## Abbreviations

The following abbreviations are used in this manuscript:

SQI	Sleep Quality Index
EEG	Electroencephalographic
EOG	Electrooculographic
PPG	Photoplethysmographic
ASI	Italian Space Agency
ECG	Electrocardiographic
RR	Respiratory Rate
DL	Deep Learning
BOAS	Bitbrain Open Access Sleep
mRMR	minimum Redundancy Maximum Relevance
PCA	Principal Component Analysis
LSTM	Long Short-Term Memory
REM	Rapid Eye Movement
NREM	Non-Rapid Eye Movement
SE	Sleep Efficiency
TST	Total Sleep Time Sleep
WASO	Wake After Sleep Onset
SFI	Sleep Fragmentation Index
IRCCS	Istituto di Ricovero e Cura a Carattere Scientifico
LOSO	Leave-One-Subject-Out
HMM	Hidden Markov Model
PSG	Polysomnography
PSQI	Pittsburgh Sleep Quality Index
PROMIS	Patient-Reported Outcomes Measurement Information System
ESS	Epworth Sleepiness Scale
SSA	Self-rating Sleep and Awakening
SL	Sleep Latency
HRV	Heart Rate Variability
AASM	American Academy of Sleep Medicine
NSF	National Sleep Foundation
SOL	Sleep Onset Latency
SWS	Slow Wave Sleep
MAI	Micro-Arousal Index
ArI	Arousal Index
NSS	Number of Stage Shifts
R	Regularity
S	Satisfaction
A	Alertness
T	Timing
E	Efficiency
D	Duration
SRI	Sleep-Related Impairment
TNR	Total NREM sleep duration
NAE	Number of Apnea Episodes
PT	Primary posture Time
DST	Deep Sleep Time
RST	REM Sleep Time
TIW	Time In Wake
ATS	Awakenings Total Score
SQS	Sleep Quality Score
IER	Inspiratory-to-Expiratory Ratio
PCs	Principal Components
EMG	Electromyographic
TQR	Total Quality Recovery
ADASYN	Adaptive Synthetic Sampling
SMOTE	Synthetic Minority Over-Sampling Technique

## References

[1] Ramar K., Malhotra R.K., Carden K.A., Martin J.L., Abbasi-Feinberg F., Aurora R.N., Kapur V.K., Olson E.J., Rosen C.L., Rowley J.A. (2021). Sleep Is Essential to Health: An American Academy of Sleep Medicine Position Statement. J. Clin. Sleep Med..

[2] Ramos A.R., Wheaton A.G., Johnson D.A. (2023). Sleep Deprivation, Sleep Disorders, and Chronic Disease. Prev. Chronic Dis..

[3] Del Rosso L.M. (2025). Global Perspectives on Sleep Health: Definitions, Disparities, and Implications for Public Health. Brain Sci..

[4] Zong H., Fei Y., Liu N. (2025). Circadian Disruption and Sleep Disorders in Astronauts: A Review of Multi-Disciplinary Interventions for Long-Duration Space Missions. Int. J. Mol. Sci..

[5] Albornoz-Miranda M., Parrao D., Taverne M. (2023). Sleep Disruption, Use of Sleep-Promoting Medication and Circadian Desynchronization in Spaceflight Crewmembers: Evidence in Low-Earth Orbit and Concerns for Future Deep-Space Exploration Missions. Sleep Med. X.

[6] Hirshkowitz M. (2014). Polysomnography: Understanding This Technology’s Past Might Guide Future Developments. IEEE Pulse.

[7] Kukwa W., Migacz E., Lis T., Ishman S.L. (2021). The Effect of In-Lab Polysomnography and Home Sleep Polygraphy on Sleep Position. Sleep Breath.

[8] Guo J.-H., Qu W.-M., Chen S.-G., Chen X.-P., Lv K., Huang Z.-L., Wu Y.-L. (2014). Keeping the Right Time in Space: Importance of Circadian Clock and Sleep for Physiology and Performance of Astronauts. Mil. Med. Res..

[9] De Fazio R., Mastronardi V.M., De Vittorio M., Spongano L., Fachechi L., Rizzi F., Visconti P. A Sensorized Face Mask to Monitor Sleep and Health of the Astronauts: Architecture Definition, Sensing Section Development and Biosignals’ Acquisition. Proceedings of the 2024 9th International Conference on Smart and Sustainable Technologies (SpliTech).

[10] De Fazio R., Cascella I., Al-Naami B., De Vittorio M., Visconti P. EEG Signal Acquisition from the Forehead and Ears through Textile-Based 3D-Printed Electrodes to Be Integrated into a Sensorized Face-Mask for Astronauts’ Sleep Monitoring. Proceedings of the 2024 Second Jordanian International Biomedical Engineering Conference (JIBEC).

[11] De Fazio R., Cascella I., Yalçınkaya Ş.E., De Vittorio M., Patrono L., Velazquez R., Visconti P. (2025). Synchronous Acquisition and Processing of Electro- and Phono-Cardiogram Signals for Accurate Systolic Times’ Measurement in Heart Disease Diagnosis and Monitoring. Sensors.

[12] Shumba A.T., Demir S.M., Mastronardi V.M., Rizzi F., De Marzo G., Fachechi L., Ros P.M., Demarchi D., Patrono L., De Vittorio M. (2024). Monitoring Cardiovascular Physiology Using Bio-Compatible AlN Piezoelectric Skin Sensors. IEEE Access.

[13] Choi J.H., Ha T.K., Moon J.E., Chung S. (2026). Analysis of Interrater Reliability and Interpretive Discrepancies in Polysomnography Scoring Across Clinical Subgroups. Life.

[14] Sarkar D., Guha D., Tarafdar P., Sarkar S., Ghosh A., Dey D. (2022). A Comprehensive Evaluation of Contemporary Methods Used for Automatic Sleep Staging. Biomed. Signal Process. Control.

[15] De Fazio R., Yalçınkaya Ş.E., Cascella I., Del-Valle-Soto C., De Vittorio M., Visconti P. (2025). Forehead and In-Ear EEG Acquisition and Processing: Biomarker Analysis and Memory-Efficient Deep Learning Algorithm for Sleep Staging with Optimized Feature Dimensionality. Sensors.

[16] López-Larraz E. (2024). Bitbrain Open Access Sleep Dataset. OpenNeuro.

[17] Lee S., Kim J.H., Chung J.H. (2021). The Association between Sleep Quality and Quality of Life: A Population-Based Study. Sleep Med..

[18] Ohayon M., Wickwire E.M., Hirshkowitz M., Albert S.M., Avidan A., Daly F.J., Dauvilliers Y., Ferri R., Fung C., Gozal D. (2017). National Sleep Foundation’s Sleep Quality Recommendations: First Report. Sleep Health.

[19] Vitale J.A., Galbiati A., Giacomi G.D., Tornese D., Levendowski D., Ferini-Strambi L., Banfi G. (2022). Sleep architecture in response to a late evening competition in team-sport athletes. Int. J. Sports Phys. Perf..

[20] Hirshkowitz M., Chokroverty S., Billiard M. (2015). The History of Polysomnography: Tool of Scientific Discovery. Sleep Medicine.

[21] Mendonça F., Mostafa S.S., Morgado-Dias F., Ravelo-García A.G., Penzel T. (2019). A Review of Approaches for Sleep Quality Analysis. IEEE Access.

[22] Pierson-Bartel R., Ujma P.P. (2024). Objective Sleep Quality Predicts Subjective Sleep Ratings. Sci. Rep..

[23] Webber H.E., Badawi J.C., Schmitz J.M., Yoon J.H., Calvillo D.J., Becker C.I., Lane S.D. (2025). Objective and Subjective Measurement of Sleep in People Who Use Substances: Emerging Evidence and Recommendations from a Systematic Review. J. Sleep Res..

[24] Carpi M. (2025). Correction to: The Pittsburgh Sleep Quality Index: A Brief Review. Occup. Med..

[25] Evans J.P., Smith A., Gibbons C., Alonso J., Valderas J.M. (2018). The National Institutes of Health Patient-Reported Outcomes Measurement Information System (PROMIS): A View from the UK. Patient Relat. Outcome Meas..

[26] Scharf M.T. (2022). Reliability and Efficacy of the Epworth Sleepiness Scale: Is There Still a Place for It?. Nat. Sci. Sleep.

[27] Snyder E., Cai B., DeMuro C., Morrison M.F., Ball W. (2018). A New Single-Item Sleep Quality Scale: Results of Psychometric Evaluation in Patients With Chronic Primary Insomnia and Depression. J. Clin. Sleep Med..

[28] Berry R.B., Albertario C., Harding S. (2018). The AASM Manual for the Scoring of Sleep and Associated Events: Rules, Terminology and Technical Specifications: Version 2.5.

[29] Wang L.-L., Zheng W.-L., Ma H.-W., Lu B.-L. Measuring Sleep Quality from EEG with Machine Learning Approaches. Proceedings of the 2016 International Joint Conference on Neural Networks (IJCNN).

[30] Hirshkowitz M., Whiton K., Albert S.M., Alessi C., Bruni O., DonCarlos L., Hazen N., Herman J., Katz E.S., Kheirandish-Gozal L. (2015). National Sleep Foundation’s Sleep Time Duration Recommendations: Methodology and Results Summary. Sleep Health.

[31] Riedy S.M., Smith M.G., Rocha S., Basner M. (2021). Noise as a Sleep Aid: A Systematic Review. Sleep Med. Rev..

[32] Wood K.H., Memon A.A., Memon R.A., Joop A., Pilkington J., Catiul C., Gerstenecker A., Triebel K., Cutter G., Bamman M.M. (2021). Slow Wave Sleep and EEG Delta Spectral Power Are Associated with Cognitive Function in Parkinson’s Disease. J. Parkinson’s Dis..

[33] Qian X., Qiu Y., He Q., Lu Y., Lin H., Xu F., Zhu F., Liu Z., Li X., Cao Y. (2021). A Review of Methods for Sleep Arousal Detection Using Polysomnographic Signals. Brain Sci..

[34] Haba-Rubio J., Ibanez V., Sforza E. (2004). An Alternative Measure of Sleep Fragmentation in Clinical Practice: The Sleep Fragmentation Index. Sleep Med..

[35] Morrell M.J., Finn L., Kim H., Peppard P.E., Badr M.S., Young T. (2000). Sleep Fragmentation, Awake Blood Pressure, and Sleep-Disordered Breathing in a Population-Based Study. Am. J. Respir. Crit. Care Med..

[36] Hachenberger J., Baron S., Schabus M., Lemola S. (2025). The Role of Objective Sleep Duration, Continuity, and Architecture for Subjective Sleep Perception: Findings from an Intensive Longitudinal Study Using Heart-Rate Variability to Infer Objective Sleep Indicators. Sleep Med..

[37] Chouraki A., Tournant J., Arnal P., Pépin J.-L., Bailly S. (2023). Objective Multi-Night Sleep Monitoring at Home: Variability of Sleep Parameters between Nights and Implications for the Reliability of Sleep Assessment in Clinical Trials. Sleep.

[38] Bonnet M.H., Arand D.L. (2007). EEG Arousal Norms by Age. J. Clin. Sleep Med..

[39] Balakrishnan G., Burli D., Behbehani K., Burk J., Lucas E. Comparison of a Sleep Quality Index between Normal and Obstructive Sleep Apnea Patients. Proceedings of the 2005 IEEE Engineering in Medicine and Biology 27th Annual Conference.

[40] Yoo A., Vgontzas A., Chung J., Mostofsky E., Li W., Rueschman M., Buysse D., Mittleman M., Bertisch S. (2023). The Association between Multidimensional Sleep Health and Migraine Burden among Patients with Episodic Migraine. J. Clin. Sleep Med..

[41] Nam Y., Kim Y., Lee J. (2016). Sleep Monitoring Based on a Tri-Axial Accelerometer and a Pressure Sensor. Sensors.

[42] Shambroom J.R., Fábregas S.E., Johnstone J. (2012). Validation of an Automated Wireless System to Monitor Sleep in Healthy Adults. J. Sleep Res..

[43] Cheng S.-P., Mei H. A Personalized Sleep Quality Assessment Mechanism Based on Sleep Pattern Analysis. Proceedings of the 2012 Third International Conference on Innovations in Bio-Inspired Computing and Applications.

[44] Miwa H., Sasahara S., Matsui T. Roll-over detection and sleep quality measurement using a wearable sensor. Proceedings of the 2007 29th Annual International Conference of the IEEE Engineering in Medicine and Biology Society.

[45] Han T.-Y., Min S.-D., Nam Y. A Real-Time Sleep Monitoring System with a Smartphone. Proceedings of the 2015 9th International Conference on Innovative Mobile and Internet Services in Ubiquitous Computing.

[46] De Fazio R., Spongano L., De Vittorio M., Patrono L., Visconti P. (2024). Machine Learning Algorithms for Processing and Classifying Unsegmented Phonocardiographic Signals: An Efficient Edge Computing Solution Suitable for Wearable Devices. Sensors.

[47] Laganà F. (2026). Design and Simulation-Based Validation of an Embedded Acquisition Architecture for In Situ PCB Integrity Monitoring in Biomedical Devices. Electronics.

[48] Coluccio M.L., Pullano S.A., Vismara M.F.M., Coppedè N., Perozziello G., Candeloro P., Gentile F., Malara N. (2020). Emerging Designs of Electronic Devices in Biomedicine. Micromachines.

[49] Supratak A., Dong H., Wu C., Guo Y. (2017). DeepSleepNet: A Model for Automatic Sleep Stage Scoring Based on Raw Single-Channel EEG. IEEE Trans. Neural Syst. Rehabil. Eng..

[50] Phan H., Andreotti F., Cooray N., Chen O.Y., De Vos M. (2019). SeqSleepNet: End-to-End Hierarchical Recurrent Neural Network for Sequence-to-Sequence Automatic Sleep Staging. IEEE Trans. Neural Syst. Rehabil. Eng..

[51] Phan H., Mikkelsen K., Chen O.Y., Koch P., Mertins A., De Vos M. (2022). SleepTransformer: Automatic Sleep Staging With Interpretability and Uncertainty Quantification. IEEE Trans. BioMed Eng..

[52] Phan H., Lorenzen K.P., Heremans E., Chén O.Y., Tran M.C., Koch P., Mertins A., Baumert M., Mikkelsen K.B., De Vos M. (2023). L-SeqSleepNet: Whole-Cycle Long Sequence Modeling for Automatic Sleep Staging. IEEE J. Biomed. Health Inform..

[53] Laganà F., Pratticò D., Quattrone M.F., Pullano S.A., Calcagno S. (2026). Hybrid AI–Taguchi–ANOVA Approach for Thermographic Monitoring of Electronic Devices. Eng.

[54] Bibbò L., Laganà F., Bilotta G., Meduri G.M., Angiulli G., Cotroneo F. (2025). AI-Enhanced Eco-Efficient UAV Design for Sustainable Urban Logistics: Integration of Embedded Intelligence and Renewable Energy Systems. Energies.

[55] Khalighi S., Sousa T., Santos J.M., Nunes U. (2016). ISRUC-Sleep: A Comprehensive Public Dataset for Sleep Researchers. Comput. Methods Programs Biomed..

[56] Mai X., Song B., Luo M., Zhu J., Jiang X., Ma X., Lin F., Hu X., Peng H., Zhang L. (2025). AISleep: Automated and Interpretable Sleep Staging from Single-Channel EEG Data. Patterns.

[57] Baumert M., Hartmann S., Phan H. (2023). Automatic Sleep Staging for the Young and the Old—Evaluating Age Bias in Deep Learning. Sleep Med..

